# Trigger Factor in *Burkholderia pseudomallei* is essential for key virulence determinants, including host cell internalization, cytotoxicity, motility, and stress resistance

**DOI:** 10.1128/jb.00124-26

**Published:** 2026-04-22

**Authors:** Justine B. Bendo, Aleksandra W. Debowski, Nicole M. Bzdyl, Jua Iwasaki, Josephine Starr, Charles S. Bond, Nichollas E. Scott, Keith A. Stubbs, Mitali Sarkar-Tyson

**Affiliations:** 1 Marshall Centre for Interventions in Infectious Disease, School of Biomedical Sciences, University of Western Australia 2720, Perth, Western Australia, Australia; 2 School of Molecular Sciences, The University of Western Australia 532399, Crawley, Western Australia, Australia; 3 Wesfarmers Centre for Vaccines and Infectious Diseases, The Kids Research Institute Australia, University of Western Australia 117610, Nedlands, Western Australia, Australia; 4 Centre for Child Health Research, University of Western Australia 2720, Perth, Western Australia, Australia; 5 Department of Microbiology and Immunology, University of Melbourne at the Peter Doherty Institute for Infection and Immunity, Parkville, Victoria, Australia; 6 ARC Training Centre for Next-Gen Technologies in Biomedical Analysis, School of Molecular Sciences, The University of Western Australia, Crawley, Western Australia, Australia; Southern University of Science and Technology, Shenzhen, Guangdong, China

**Keywords:** Trigger Factor, *Burkholderia pseudomallei*, virulence, cell infection, FK506-binding proteins

## Abstract

*Burkholderia pseudomallei* is a facultative intracellular pathogen and the causative agent of melioidosis. Treatment of this deadly infection is both protracted and limited to only a select number of antibiotics. Not only can patients suffer adverse reactions to prolonged therapy, but resistance has also been reported in several clinical isolates. Therefore, it is essential to identify new drug targets to combat this infection. One approach is to identify and target proteins that are involved in bacterial virulence. Trigger Factor (TF) belongs to the FK506-binding protein family and functions as both a peptidyl-prolyl *cis-trans* isomerase and as a chaperone. TF has been shown to be important for virulence in gram-positive bacteria. However, very limited information is available about the contribution of TF to virulence in *B. pseudomallei* or other pathogenic gram-negative bacteria. In this study, the *B. pseudomallei* TF homolog (BPSL1402) was enzymatically characterized, and a deletion mutant was generated to investigate the role of TF in virulence. Comparative proteomic analysis coupled with cell-based infection and phenotypic assays revealed that deletion of *tig* in *B. pseudomallei* resulted in pleiotropic effects on multiple virulence mechanisms. The *Bp∆tig* mutant was highly attenuated in cells, showing defects in adhesion and internalization, as well as reduced cell cytotoxicity. Furthermore, the mutant demonstrated altered capsule and lipopolysaccharide production, increased biofilm formation, reduced motility, and increased susceptibility to oxidative and pH stresses. The results in this study indicate that *tig* is important for the virulence of *B. pseudomallei* and is a potential target for anti-virulence therapy.

**IMPORTANCE:**

Melioidosis is a potentially fatal infection caused by *Burkholderia pseudomallei*, a bacterium that is intrinsically resistant to many commonly used antibiotics. Therefore, the identification of new drug targets is essential for the development of new and effective therapies. This study demonstrates that the Trigger Factor protein, encoded by BPSL1402, is important for the establishment of *B. pseudomallei* infection and is involved in multiple facets of virulence, including motility, cell cytotoxicity, and resistance to stress. With reports of *B. pseudomallei* isolated from new regions and resistance to current treatment options, the significance of this research is the identification of a novel *B. pseudomallei* virulence factor that can be potentially exploited for the development of new therapeutics to treat this deadly infection.

## INTRODUCTION

Melioidosis is a potentially fatal disease caused by the multi-drug-resistant gram-negative bacteria, *Burkholderia pseudomallei* (1). This organism is endemic in the soil and waterways of tropical and subtropical areas, particularly in Southeast Asia and Northern Australia. However, with increased surveillance and improved bacterial genotyping, new areas of endemicity have recently been established, including the Mississippi Gulf Coast and South East Queensland (2, 3). *B. pseudomallei* infection is acquired through environmental exposure to contaminated soil and water, predominantly through subcutaneous inoculation or infection of open wounds, inhalation of infectious particles, as well as ingestion of contaminated food or water (4). *B. pseudomallei* has earned the title of “The Great Mimicker” as the clinical presentation of melioidosis is highly variable, with symptoms ranging from skin abscesses and pneumonia to more severe manifestations such as septicemia and neurological melioidosis (4, 5). Evidence-based modeling estimates the global number of deaths due to melioidosis to be approximately 89,000 deaths per annum (6). However, this may be an underestimation due to misdiagnosis and lack of awareness of the disease (6–9). The mortality rates of melioidosis vary among different countries and can range from ~10%–15% in Australia to 40% in northeast Thailand despite intensive antibiotic interventions (10–12).

Treatment of melioidosis relies on a limited number of antibiotics, including ceftazidime or meropenem for the initial intensive therapy phase, and oral trimethoprim-sulfamethoxazole or doxycycline for subsequent eradication therapy (13). Worryingly, recent reports have described several clinical *B. pseudomallei* isolates with resistance to ceftazidime and meropenem (14, 15). Furthermore, antibiotic treatment may often need to be changed or temporarily withdrawn due to adverse reactions, such as acute kidney injury, severe cutaneous adverse drug reactions, and bone marrow suppression, experienced by the patient (16). Therefore, new therapeutic targets need to be identified to develop effective treatment options for *B. pseudomallei* infection.

As a facultative intracellular pathogen, *B. pseudomallei* encodes multiple virulence determinants, allowing the bacterium to survive intracellularly and evade the host’s immune response (1). Attachment and adhesion of *B. pseudomallei* to host cells have been shown to be influenced by the flagella (17), trimeric autotransporters such as the *Burkholderia* Oca-like Adhesin A proteins (BoaA and BoaB) (18–20), and the *Burkholderia* Surface Attachment Protein 1 (Sap1) (21, 22). Upon phagocytosis by the host cells, the bacteria employ several stress responses through the action of multiple Sigma Factors, including RpoS, RpoH, and RpoN to regulate stress response mechanisms (23–25), catalase proteins KatE and KatG to directly inhibit oxidative stress response, and various DNA-binding proteins that protect the bacterium against direct DNA damage (26, 27), all allowing the bacterium to survive host-cell killing mechanisms. *B. pseudomallei* also employs its Type III secretion system, particularly secretion of the protein *Burkholderia* invasion protein D (BipD), to rapidly escape the phagolysosome and enter the cell cytosol, where it starts to replicate (28). Inside the host cell cytosol, the *Burkholderia* intracellular motility (Bim) proteins are used to hijack host actin to form bacterial tails, facilitating intracellular movement (29, 30). When *B. pseudomallei* reaches the host cell membrane, the bacteria utilize their Type VI secretion system (T6SS), particularly the hemolysin-coregulated protein 1 (Hcp1), to induce the fusion of adjacent cells, resulting in the formation of a multinucleated giant cell (MNGC). This mechanism has been shown to be essential for *in vivo* virulence and immune evasion (31). As the infection progresses, the host cell can lyse, releasing the bacteria into the extracellular space and perpetuating the infection.

Previous studies have shown that peptidyl-prolyl *cis-trans* isomerases (PPIases), such as the Macrophage infectivity potentiator (Mip) and cyclophilin B, are also important for virulence and the intracellular lifecycle of *B. pseudomallei* (32, 33). PPIases facilitate the isomerization of proline bonds in their target proteins, a recognized rate-limiting step in protein folding. Additionally, some PPIases exhibit chaperone activity (34, 35). Importantly, PPIases are druggable and can be inhibited by natural products such as the immunosuppressants FK506 (tacrolimus), rapamycin (sirolimus) (34), and Cyclosporin A (36). Furthermore, simpler, non-immunosuppressive pipecolic acid-based compounds targeting Mip have been shown to significantly reduce the virulence of *B. pseudomallei* and suppress damaging pro-inflammatory immune responses *in vitro* (37–39). Therefore, with the aim of identifying additional targets for therapeutic intervention against *B. pseudomallei* infection, it is important to investigate whether other PPIases in *B. pseudomallei* also contribute to its virulence.

This study investigated the PPIase, Trigger Factor (TF), which, like Mip, belongs to the FK506-binding protein (FKBP) family. TF is the primary ribosome-associated chaperone in bacteria, and its molecular and structural function has been well characterized in *Escherichia coli* models (40, 41). TF is composed of three domains: an N-terminal ribosomal binding domain, a central PPIase domain, and a C-terminal putative chaperone domain, which, together with the N-terminal domain, forms a cradle that interacts with the nascent unfolded proteins exiting the ribosome (42, 43).

Several studies have shown that TF is involved in multiple virulence mechanisms in gram-positive bacteria, including *Listeria monocytogenes* (44), *Staphylococcus aureus* (45), and *Streptococcus* spp. (46, 47). To date, there is very limited information about the role of TF in the virulence of pathogenic gram-negative bacteria. In *E. coli*, the deletion of *tig* (gene encoding Trigger Factor) did not result in any major alterations in growth (48). However, the potential role of *tig* in virulence has not yet been directly investigated. One study in *Sinorhizobium meliloti,* a gram-negative soil microbe, reported that deletion of *tig* resulted in decreased salt tolerance and reduced competitiveness in nodule occupancy (49). Therefore, it is essential to gain a greater understanding of the functional role of TF in gram-negative pathogens.

Within this study, the role of the *B. pseudomallei* TF homolog (*Bp*TF), encoded by BPSL1402 (*tig*), in virulence was investigated. A *tig* deletion mutant in the clinical *B. pseudomallei* strain K96243 (*Bp∆tig*) was constructed and characterized using cell-based infection assays, comparative proteomic analysis, as well as growth and stress assays. It is shown that the loss of *tig* in *B. pseudomallei* led to significant dysregulation of the proteome and resulted in altered virulence phenotypes, including attenuated entry into host cells, reduced host cell cytotoxicity, changes in polysaccharide biosynthesis and biofilm formation, as well as reduced motility and increased sensitivity to stress. This study highlights the importance of *tig* in *B. pseudomallei* virulence, revealing the involvement of TF in multiple bacterial virulence determinants.

## RESULTS

### *Bp∆tig* is attenuated *in vitro* but persists longer in host cells

**Fig 1:**
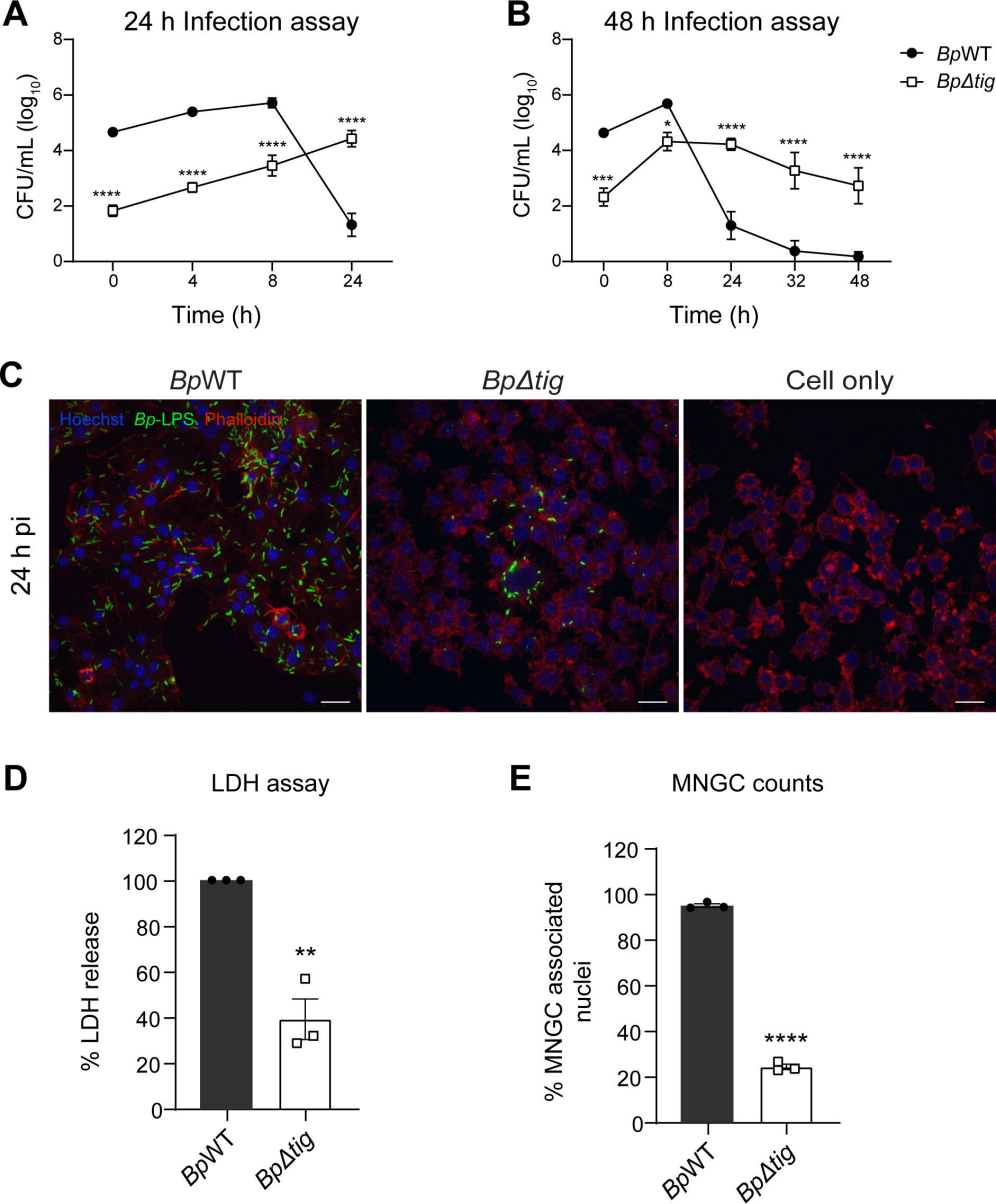
The intracellular replication, cytotoxicity, and MNGC formation of the *Bp∆tig* deletion mutant. (**A and B**) RAW264.7 cells were infected with the parent *Bp*WT (closed circles) or the *Bp∆tig* (open squares) mutant strain for 30 min at a multiplicity of infection of 10. The extracellular bacteria were killed with kanamycin, and the intracellular bacteria were enumerated at the indicated time points over (**A**) 24 h (*n* = 3) or (**B**) 48 h (*n* = 3). Data are presented as log_10_ CFU/mL, with error bars representing the standard error of the mean. *, *P* < 0.05; **, *P* < 0.01. *P* values were determined using multiple unpaired *t*-test. (**C**) Representative confocal fluorescence images of infected RAW264.7 cells at 24 h pi. Cell nuclei were stained with Hoechst 33258 (blue), bacteria with anti-*B. pseudomallei*-lipopolysaccharide (green), and host cell actin with phalloidin (red). Scale bar, 20 µm. (**D**) The lactate dehydrogenase (LDH) release in the supernatant by cells infected with *Bp*WT (closed circles) or *Bp∆tig* (open squares) after 24 h pi (*n* = 3). Error bars represent the standard error of the mean. **, *P* = 0.0024. *P* values were determined using one-sample *t*-test. (**E**) The percentage of nuclei associated with MNGCs in the *Bp*WT (closed circles) and *Bp∆tig* (open squares) infected monolayers (*n* = 3). Error bars represent the standard error of the mean. ****, *P* < 0.0001. *P* values were determined using an unpaired *t-*test.

To investigate the role of *tig* in *B. pseudomallei* virulence, an in-frame unmarked *tig* deletion mutant in *B. pseudomallei* strain K96243 was constructed using the *sacB* counter-selection method described by Logue et al. (50), producing the mutant *Bp∆tig*. Growth curves conducted in Luria-Bertani (LB) broth showed that the *Bp∆tig* deletion mutant had no apparent growth defect compared to the parent strain (Fig. S1). To investigate if *tig* was required for virulence in *B. pseudomallei*, RAW 264.7 murine macrophage cells were infected with *Bp*WT or *Bp∆tig,* and the number of intracellular bacteria was enumerated over time. Interestingly, the number of bacteria recovered at 0, 4, and 8 h post-infection (pi) was significantly lower for the *Bp∆tig* strain compared to *Bp*WT, indicating that *tig* is important for the uptake of *B. pseudomallei* into cells (Fig. 1A). At 24 h pi, the number of recoverable colony-forming units (CFUs) for the *Bp*WT strain was substantially lower compared to 8 h pi. This result was expected as *B. pseudomallei* is cytotoxic and can lyse host cells over time. This leads to the release of intracellular bacteria into the extracellular environment, where they are killed by the high levels of kanamycin in the culture medium, leading to the observed reduction in the recoverable CFU. In contrast, the number of recoverable CFU for the *Bp∆tig* mutant was higher at 24 h pi compared to 8 h pi, suggesting that the mutant strain was not lysing the host cells. Since the intracellular counts of the *Bp∆tig* mutant continued to increase over 24 h, the infection was extended to 48 h to investigate if the mutant strain could achieve the same peak intracellular counts observed for the *Bp*WT strain at 8 h pi. Again, the number of recoverable CFU for the *Bp*WT strain sharply decreased after 8 h pi and continued to decline from 24 to 48 h pi (Fig. 1B). In contrast, for the *Bp∆tig* mutant, the number of recoverable CFU plateaued after 8 h pi and slowly decreased from 24 to 48 h pi. The number of intracellular *Bp∆tig* bacteria remained significantly higher than the parent strain at these later time points.

The attenuation of the *Bp∆tig* mutant, observed through intracellular counts, was supported by fluorescence imaging of infected RAW monolayers at 24 h pi. Monolayers infected with *Bp*WT showed widespread bacterial infiltration of host cells and the characteristic formation of multinucleated giant cells (MNGCs) (Fig. 1C, panel 1). In contrast, fluorescence images of *Bp∆tig*-infected cells revealed an intact monolayer (Fig. 1C, panel 2) that looked similar to the uninfected cell control (Fig. 1C, panel 3). The *Bp∆tig* cells remained localized within the RAW264.7 cells, and there was reduced MNGC formation.

To further investigate whether the *Bp∆tig* mutant was less cytotoxic than *Bp*WT, the amount of lactate dehydrogenase (LDH) in the supernatant was quantified. LDH is released upon cell death and has been used to quantify the cytotoxicity of *B. pseudomallei* to host cells (37, 38). As expected, lower levels of LDH were measured in the supernatant of *Bp∆tig*-infected monolayers at 24 h pi relative to the LDH released by *Bp*WT-infected cells (set to 100%) (Fig. 1D). In addition, the level of MNGC formation was quantified by enumerating the nuclei associated with MNGCs. Only 24.6% of host cell nuclei were associated with MNGCs in the cell monolayer infected with *Bp∆tig* compared to 94.2% in monolayers infected with *Bp*WT (Fig. 1E). Taken altogether, these results show that *Bp∆tig* is attenuated *in vitro* but can persist inside host cells for up to 48 h post-infection.

### Proteomics analysis of the *Bp∆tig* mutant reveals significant disruption of virulence-associated proteins

**Fig 2:**
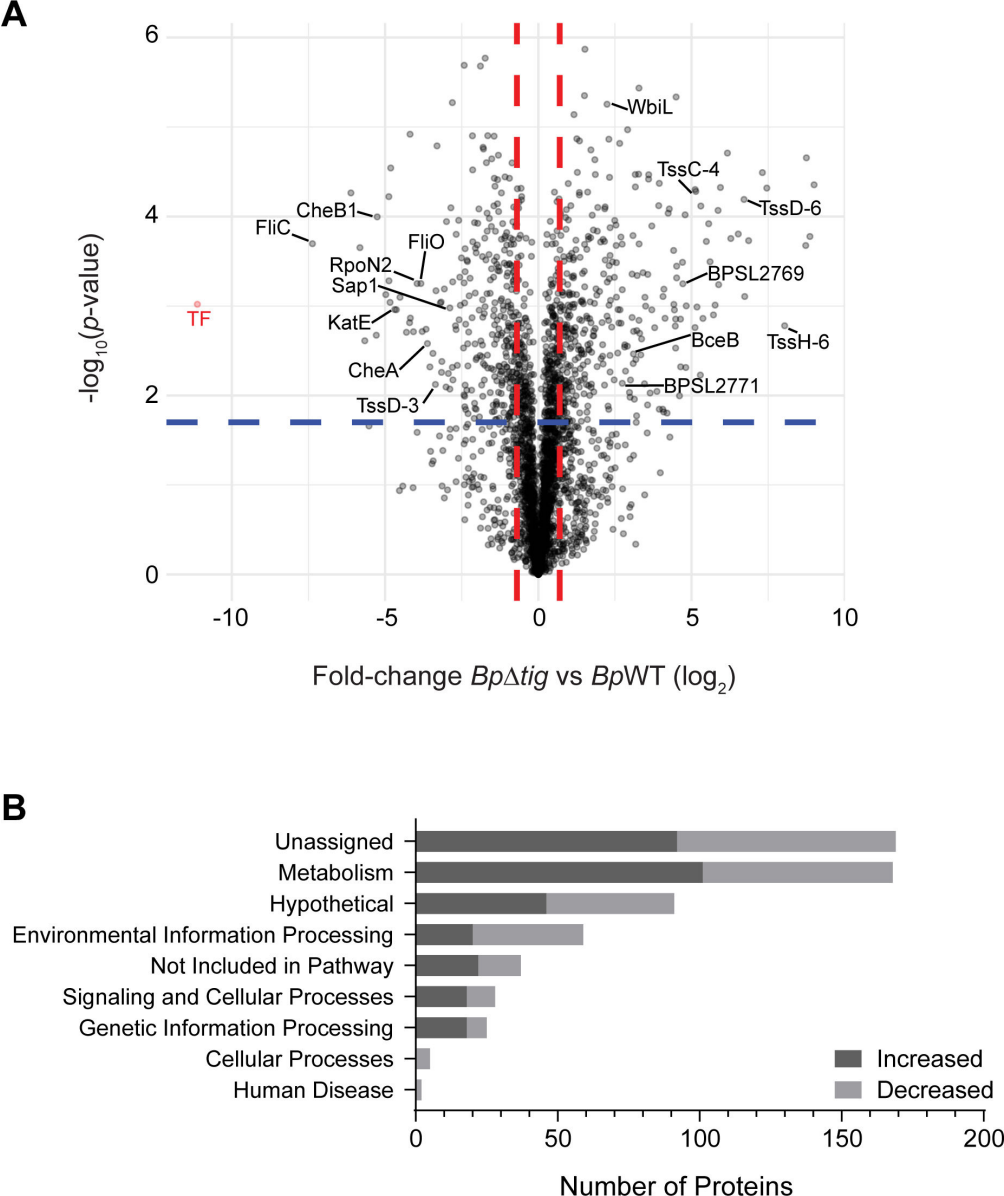
Quantitative proteomic analysis of *Bp*WT versus the *Bp∆tig* mutant strain. (**A**) Identified proteins are presented as a volcano plot depicting mean label-free quantitation intensity ratios of the *Bp∆tig* strain versus *Bp*WT plotted against logarithmic *t* test *P*-values from four biological replicates of each strain. (**B**) KEGG pathway analysis of differentially present proteins identified in the proteome of *Bp∆tig* compared to *Bp*WT. The KEGG database was used to manually curate the differentially present proteins in the proteome of *Bp∆tig* mutant against the *Burkholderia pseudomallei* K96243 genome (entry number T00203) and assigned a KO. Proteins that were increased in the proteome of *Bp∆tig* are represented by the dark gray bars, while the proteins identified as decreased in relative abundance are represented by light gray bars. For proteins included in multiple KOs, only the first annotation was recorded.

To gain insight into the proteomic changes within *Bp∆tig* influencing the loss of virulence, label-free-based quantitative (LFQ) proteomics was undertaken. Across *Bp∆tig* and *Bp*WT strains, a total of 3,051 proteins were identified with high consistency observed across biological replicates, as determined by Pearson correlations (average, 0.95) (Fig. S2). A total of 442 proteins were identified as altered, defined as possessing an adjusted *P*-value of ≤ 0.01 and log_2_ fold-change of <−1 and >1, with 248 and 193 increased and decreased in abundance, respectively (Fig. 2A, volcano plot; Table S1). Consistent with the deletion of the *tig* gene in strain *Bp∆tig*, BPSL1402 (*Bp*TF) showed the largest negative-fold difference (−11.13 log_2_; −log_10_[*P-*value], 3.01) within the proteome. The Kyoto Encyclopedia of Genes and Genomes (KEGG) database was used to uncover the functional pathways affected by the loss of *tig* (Fig. 2B). For proteins with multiple KEGG Orthology (KO) annotations, only the first annotated KO was recorded. Proteins annotated as “unassigned KO” formed the largest group, where 130 proteins were differentially present. Metabolism formed the second largest group, accounting for 126 differentially present proteins, with 79 being increased and 47 decreased in relative abundance. In addition, many hypothetical proteins and proteins involved in environmental information processing were also differentially present.

Closer inspection revealed some interesting changes (Table 1). First, an important adhesion protein Sap1 (−2.89 log_2_; −log_10_[*P-*value], 2.98) and the *Burkholderia* Lethal Factor 1 (−1.42 log_2_; −log_10_[*P-*value], 2.79) were decreased in abundance, and many Type VI secretion system proteins were differentially present, which may explain why the *Bp∆tig* mutant was attenuated *in vitro*. Interestingly, many T6SS-2 and T6SS-3 components were significantly increased in relative abundance, suggesting that the normally tight regulation of these T6SS has been lost or induced as a stress response. Furthermore, multiple proteins associated with stress responses, such as RpoN2 (−3.95 log_2_; −log_10_[*P-*value], 3.25) and KatE (−4.63 log_2_; −log_10_(*P-*value], 2.96), were also decreased in abundance, suggesting that the strain would be more sensitive to various environmental as well as intracellular stresses.

**TABLE 1:** List of virulence-related proteins identified as differentially present in the proteome of the *Bp∆tig* mutant compared to *Bp*WT^*b*^

Protein code	Gene code in K96243	Gene product	Fold change (log_2_)	−log10 Student’s *t*-test (*P*-value)	Ref.
Host adhesion-related proteins					
Sap1	BPSL0097	Surface attachment protein	−2.88559	2.97837	(21)
Type III secretion—T3SS-3					
BsaO	BPSS1545	Type III secretion system protein	−1.83617	2.43261	(51)
Other virulence-related proteins					
Blf1	BPSL1549	*Burkholderia* Lethal Factor 1	−1.42423	2.78902	(52, 53)
Type VI secretion—T6SS-2					
GvmR	BPSL0117	LysR family transcription regulatory protein	1.43149	3.06398	
ExbB	BPSS0367	Putative bipolymer transport protein	1.576	2.88674	
TssH-6	BPSS0516	Uncharacterized protein	4.5145	3.3142	
TssG-6	BPSS0517	Uncharacterized protein	5.14039	4.27972	
TssB-6	BPSS0522	Putative ATPase	3.70423	3.89481	
TssM-4	BPSS0532	Type VI secretion system protein ImpL	2.81749	2.54071	
Type VI secretion—T6SS-3					
TssI-6	BPSS2093	Type VI secretion system-secreted protein VgrG	4.80973	2.31268	
TssH-6^*a*^	BPSS2094	Putative Clp protease ATPase	8.03633	2.77986	
TssF-6	BPSS2096	Type VI secretion system protein ImpG	3.34122	2.45372	
TssD-6	BPSS2098	Uncharacterized protein	6.71256	4.19057	(31)
TssC-6	BPSS2099	Type VI secretion system protein ImpC	6.28031	3.75453	
TssB-6	BPSS2100	Type VI secretion system protein ImpB	6.73495	3.10557	
TssA-6	BPSS2101	Type VI secretion system protein ImpA	5.86334	4.06783	
TagE-6	BPSS2102	Non-specific serine/threonine protein kinase	5.7691	3.01038	
TssM-6	BPSS2104	Type VI secretion system protein ImpL	6.52145	3.80516	
TssK-6	BPSS2106	Type VI secretion system protein ImpJ	4.47913	2.52936	
TssJ-6	BPSS2107	Type VI secretion system protein VasD	4.42248	3.01837	
TagI-6	BPSS2108	Putative lipoprotein	4.82678	3.00185	
TagH-6	BPSS2109	FHA domain-containing protein	5.37294	2.86093	
Type VI secretion—T6SS-4					
Hcp4	BPSS0171	Type VI secretion system-secreted protein Hcp	−3.35878	2.12377	(31)
Type VI secretion—T6SS-6					
Hcp6	BPSL3105	Type VI secretion system-secreted protein Hcp	−1.43748	3.50556	(31)
TagM-1	BPSL3108	Putative lipoprotein	−1.25011	2.23589	
Stress response					
	BPSL0808	Periplasmic serine endoprotease DegP-like	−1.71172	3.51487	(54)
SodB	BPSL0880	Superoxide dismutase	−1.03646	2.2399	(55)
ClpP	BPSL1403	ATP-dependent Clp protease, protease subunit	−1.69288	4.60235	
KatE	BPSS2214	Catalase	−4.63093	2.95897	(56)
RpoN2	BPSS2218	RNA polymerase sigma-54 factor	−3.95403	3.25012	
Motility and chemotaxis					
FliL	BPSL0026	Flagellar basal body protein	−3.15792	2.25893	
FliO	BPSL0029	Flagellar protein	−3.83194	3.25118	
	BPSL2367	Putative methyl-accepting chemotaxis protein	−4.07566	2.71206	
CheZ	BPSL3299	Protein phosphatase CheZ	−1.03849	2.43367	
CheB1	BPSL3301	Chemotaxis response regulator protein-glutamate methylesterase 1	−5.25814	3.99665	
CheW	BPSL3305	Chemotaxis protein	−3.79083	2.71497	
CheA	BPSL3306	Chemotaxis two-component sensor kinase CheA	−3.62115	2.5804	
FliC	BPSL3319	Flagellin	−7.3727	3.69608	(57)
	BPSL3338	Putative methyl-accepting chemotaxis protein	−2.54471	3.00786	
Tar protein	BPSS0215	Methyl-accepting chemotaxis protein	−4.51532	3.09694	
Lipopolysaccharide biosynthesis					
WbiL	BPSL2672	Putative epimerase/dehydratase polysaccharide-related biosynthesis protein	2.24222	5.25517	(54)
WbiA	BPSL2680	Putative O-antigen acetylase	1.1516	3.54005	(54)
Wzt	BPSL2681	ABC transporter, ATP-binding component	1.11462	3.13427	(54)
Wzm	BPSL2682	Transport permease protein	1.19847	2.39948	
RmlA	BPSL2685	Glucose-1-phosphate thymidylyltransferase	1.00482	2.11581	
Capsular polysaccharide—accessory proteins					
	BPSL2769	UTP-glucose-1-phosphate uridylyltransferase	4.71427	3.24862	
	BPSL2770	Putative capsule expression protein	3.08939	3.78693	
	BPSL2771	Putative haloacid dehalogenase-like hydrolase protein	3.00949	2.17345	
KdsA2	BPSL2772	2-dehydro-3-deoxyphosphooctonate aldolase 2	1.83376	3.02849	
	BPSL2774	Uncharacterized protein	3.30639	2.69442	
	BPSL2780	Putative capsular polysaccharide transport protein	2.39296	2.76455	
Capsular polysaccharide					
WcbS/LpxC	BPSL2788	UDP-3-O-acyl-N-acetylglucosamine deacetylase	−1.14036	2.95661	
GmhA	BPSL2795	Phosphoheptose isomerase	−1.20416	3.74495	
WcbL	BPSL2796	Putative sugar kinase	−1.29638	3.43478	
WcbK	BPSL2797	Putative GDP sugar epimerase/dehydratase protein	−1.0872	4.44284	
Capsular polysaccharide III/Type IV O-PS					
BceG	BPSS1829	Putative glycosyltransferase	1.10029	2.72865	
BceF	BPSS1830	Putative exopolysaccharide biosynthesis-related tyrosine-protein kinase	1.09932	3.32544	(58)
BceE	BPSS1831	Putative exopolysaccharide biosynthesis-related polysaccharide lipoprotein	1.20217	2.67069	
Udg2	BPSS1833	UDP-glucose 6-dehydrogenase	1.27391	4.49286	
BceB	BPSS1834	Putative lipopolysaccharide biosynthesis-related protein	2.90289	2.55069	
Capsular polysaccharide II/Type III O-PS					
	BPSS0424	Putative glycosyl transferase	−1.0282	3.45854	
	BPSS0425	Putative heptosyltransferase (O-antigen-related)	−1.56999	2.03295	
	BPSS0427	Putative O-acetyl transferase (O-antigen-related)	−2.1758	2.05927	
	BPSS0428	Putative glycosyl transferase (O-antigen-related)	−1.33469	4.14282	

^*a*^ Also associated with stress response.

^*b*^ White cells indicate relative increase; gray cells indicate relative decrease in protein abundance compared to *Bp*WT.

Several proteins associated with the motility and chemotaxis were identified as reduced in abundance, including FliC (−7.37 log_2_; −log_10_[*P-*value], 3.70), FliO (−3.83 log_2_; −log_10_[*P-*value], 3.25), and CheB1 (−5.26 log_2_; −log_10_[*P-*value], 3.40), which suggested that the *Bp∆tig* mutant may have a defect in motility. Proteins involved in the lipopolysaccharide biosynthesis clusters, exopolysaccharide biosynthesis, and capsular polysaccharide biosynthesis were significantly differentially present in the proteome of the *Bp∆tig* mutant, which may affect other virulence characteristics such as biofilm formation, and lipopolysaccharide and capsule production.

To corroborate the effects of the dysregulated proteome of the *Bp∆tig* mutant, the mutant was subjected to further phenotypic characterization to gain a better understanding of how the loss of *tig* affected *B. pseudomallei* virulence.

### The *Bp∆tig* mutant is defective in adherence and internalization

**Fig 3:**
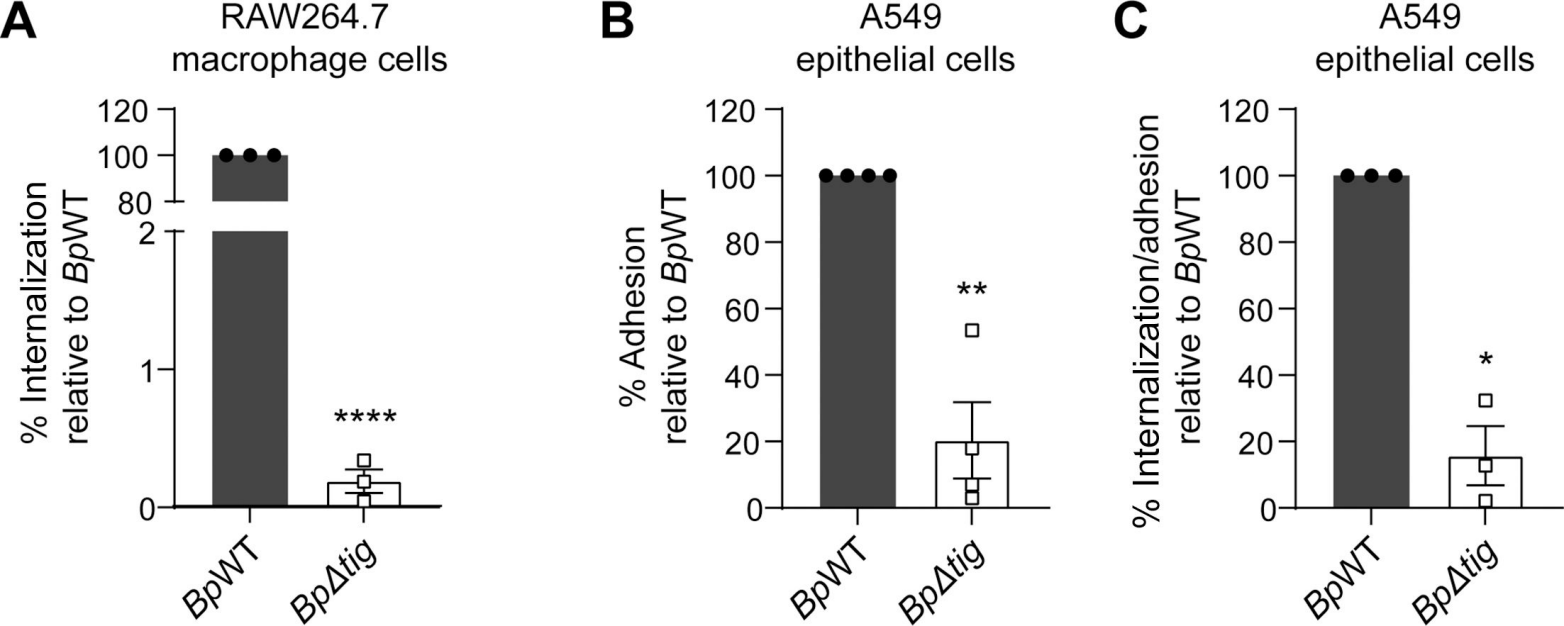
The *Bp∆tig* deletion mutant is defective in both internalization and adhesion to host cells. (**A**) RAW264.7 murine macrophage cells were infected with the *Bp*WT parent (closed circle) or the *Bp∆tig* mutant strain (open square) at a multiplicity of infection (MOI) of 10 for 30 min. Following a 30 min kill step at 0 h pi, the cells were lysed, and the supernatant was plated out to determine viable CFU. The internalization rate of the *Bp∆tig* mutant was calculated as the number of intracellular bacteria recovered (CFU) at 0 h pi divided by the bacteria in the inoculum used to infect cells (CFU), relative to *Bp*WT (*n* = 3). Error bars represent the standard error of the mean. (B and C) Initial infection of A549 lung epithelial cells. (**B**) A549 cells were infected with *Bp*WT or *Bp∆tig* at an MOI of 10 for 1 h. The infected cells were washed and then lysed, and the supernatant was plated out to determine the number of CFUs. The adhesion rate of the *Bp∆tig* mutant was calculated as the number of adhered bacteria (CFU) recovered after the infection step divided by the number of bacteria in the infecting inoculum, relative to *Bp*WT (*n* = 4). Error bars represent the standard error of the mean. (**C**) A549 cells were first infected as above but then followed with a 30 min kill step before plating out lysates for viable CFU at 0 h pi. The internalization rate of the *Bp∆tig* mutant was calculated as the number of intracellular bacteria (CFU) recovered at 0 h pi divided by the number of adherent bacteria, relative to *Bp*WT (*n* = 3). Error bars represent the standard error of the mean. *, *P* < 0.05; **, *P* < 0.01; and ****, *P* < 0.0001. *P* values were determined using one-sample *t*-test.

Since the proteomic data identified that the abundance of the adhesin Sap1 was significantly decreased in the *Bp∆tig* mutant, further work was undertaken to understand why the number of *Bp∆tig* bacteria recovered from RAW264.7 at 0 h pi was so low compared to WT. Thus, the adhesion and internalization capability of these strains was investigated using RAW264.7 and A549 cells. RAW264.7 cells are phagocytic cells and are routinely used to investigate the internalization of *B. pseudomallei* to host cells (17, 59). The rate of bacterial internalization by RAW264.7 cells was calculated for both *Bp*WT and the *Bp∆tig* mutant strains and was determined by dividing the number of intracellular bacteria recovered at 0 h pi by the number of bacteria in the inoculum used to infect the cells. The internalization rate of *Bp∆tig* was significantly reduced with only 0.19% of the mutant strain internalized by macrophage cells, relative to the parent strain (set at 100%) (Fig. 3A). These data suggest that *tig* is essential for the internalization of *B. pseudomallei* into host cells. To investigate this defect further, adherence and internalization assays were conducted using the non-phagocytic A549 lung epithelial cell line, which has previously been used to investigate the adhesion of *B. pseudomallei* to host cells (20). After 1 h of infection, only 20.3% of the *Bp∆tig* mutant adhered to A549 cells relative to the parent strain (set at 100%) (Fig. 3B). For the A549 cell internalization assays, percent internalization was calculated as the fraction of adherent cells that were internalized at 0 h pi relative to *Bp*WT. This was determined by dividing the number of intracellular bacteria recovered at 0 h pi by the number of adherent bacteria. Internalization of the *Bp∆tig* mutant strain into A549 cells was significantly reduced, with only 15.7% of the adherent bacteria internalized relative to *Bp*WT (set to 100%) (Fig. 3C). These data demonstrate that *tig* is essential for both the adhesion and the internalization of *B. pseudomallei* into host cells.

### Deletion of *tig* results in reduced bacterial motility

**Fig 4:**
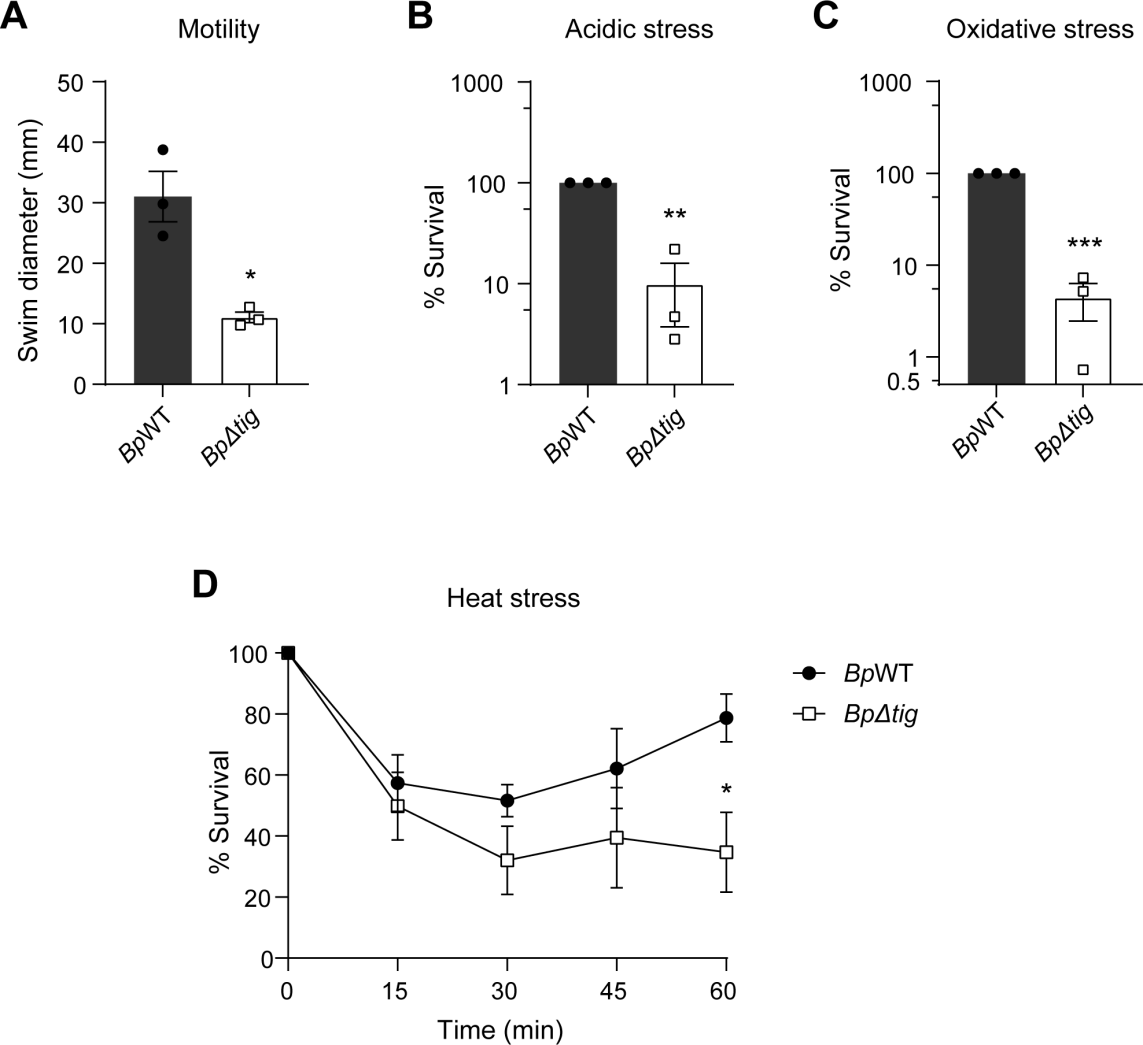
The *Bp∆tig* mutant (open squares) is less motile and more susceptible to acidic, oxidative, and heat stress compared to *Bp*WT (closed circles). (**A**) Deletion of *tig* leads to a reduction in swimming motility. Bacterial cultures were grown overnight and used to inoculate the center of 0.3% motility agar plates. Inoculated plates were incubated upright at 37°C for 24 h, after which the swim diameter of each strain was measured (*n* = 3). Error bars represent the standard error of the mean. *, *P* = 0.0358. *P* values were determined using an unpaired *t*-test. (**B**) Acidic stress assay showing decreased survival of *Bp∆tig* in pH 4. Mid-log cultures were diluted 1:100 into citrate-phosphate buffer (pH 4) and incubated at 37°C for 1 h. Data are presented as percent survival relative to *Bp*WT (*n* = 3). Error bars represent the standard error of the mean. **, *P* = 0.0046*. P* values were determined using one-sample *t*-test. (**C**) Oxidative stress assay showed increased susceptibility of *Bp∆tig* to 1 mM hydrogen peroxide. Mid-log cultures were diluted 1:2 and incubated in the presence of 1 mM H_2_O_2_ for 1 h at 37°C. Data are presented as percent survival relative to *Bp*WT, with error bars representing the standard error of the mean (*n* = 3). ***, *P* = 0.0004. *P* values were determined using one-sample *t*-test. (**D**) *Bp∆tig* is more susceptible to heat stress. Overnight cultures were standardized to an absorbance of 0.17 at 580 nm and then incubated at 50°C. The heated cultures were sampled at 15, 30, 45, and 60 min of incubation to enumerate the CFU/mL. Data are presented as percent survival relative to the starting CFU/mL, with error bars representing the standard error of the mean (*n* = 5). *, *P* = 0.0205. *P* values were determined using multiple unpaired *t*-test.

Motility has previously been reported to be important for *B. pseudomallei* entry into eukaryotic cells (17). The proteomic data revealed that the *Bp∆tig* mutant had a significant reduction in the abundance of flagella and chemotaxis-related proteins. To investigate if this reduction culminated in a motility defect, the *Bp∆tig* mutant strain was assessed for the ability to swim through 0.3% motility agar. The *Bp∆tig* mutant was observed to have a significant reduction in motility, with an average swim diameter of 11.05 mm compared to an average of 31.02 mm for the *Bp*WT parent strain (Fig. 4A).

### *tig* is required for the stress resistance response of *B. pseudomallei*

FKBPs have been reported to play a role in resistance to various stresses in several pathogenic bacteria (35). As the proteomic data of the *Bp∆tig* mutant revealed a dysregulation of multiple stress response proteins, the role of *Bp*TF in resistance to stress was investigated. The *Bp*WT parent and the *Bp∆tig* mutant strains were exposed to acidic stress (pH 4.0), oxidative stress (1 mM H_2_O_2_), and heat stress (50°C). The *Bp∆tig* mutant strain was more susceptible to acidic stress, with an average survival of only 9.9% when exposed to pH 4 for 1 h compared to the parent strain (Fig. 4B). The *Bp∆tig* mutant was also highly susceptible to oxidative stress, with an average survival of only 4.4% after exposure to 1 mM H_2_O_2_ relative to the parent strain (Fig. 4C). Finally, the strains were exposed to heat stress for up to 60 min. The *Bp∆tig* mutant strain was more sensitive to heat stress than the parent strain, but the results were not statistically significant at 30 and 45 min. However, after 45 min, the parent strain started to show signs of recovery or adaptation, unlike the mutant strain. Therefore, *Bp∆tig* showed a significant reduction in survival after 60 min of heat exposure compared to *Bp*WT (Fig. 4D). Together, these results support that *tig* is required by *B. pseudomallei* for stress resistance.

### The loss of *tig* in *B. pseudomallei* leads to a hyper-mucoid colony type and increased biofilm formation

**Fig 5:**
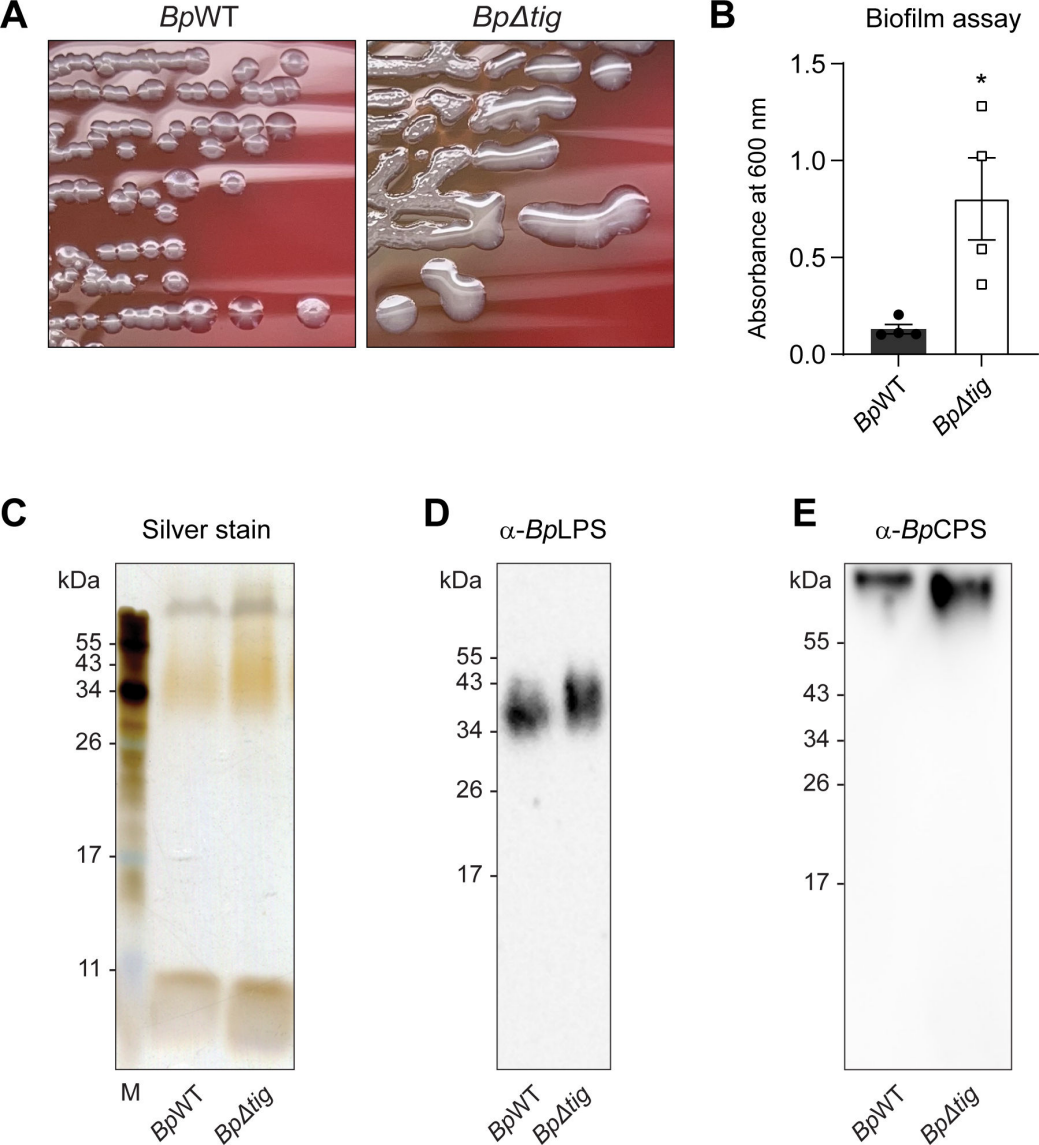
The colony morphology, biofilm-forming capacity, and polysaccharide analysis of the *Bp∆tig* mutant. (**A**) The *Bp*WT parent and *Bp∆tig* mutant strains were streaked onto CBA plates, incubated at 37°C for 48 h, and then photographed. (**B**) Biofilm-forming capacity of the *Bp*WT (closed circles) and *Bp∆tig* (open squares) strains. Biofilms were cultured for over 48 h, fixed, and then stained with crystal violet. The crystal violet was solubilized with glacial acetic acid (33%). Data are presented as the average absorbance at 600 nm (*n* = 4). Error bars represent the standard error of the mean. *, *P* = 0.0199. *P* values were determined using an unpaired *t*-test. (**C–E**) *Bp∆tig* may be producing more LPS and capsular polysaccharide (CPS). (**C**) Silver-stained SDS-PAGE gel of proteinase K-treated whole cell extracts of *Bp*WT and *Bp∆tig*. Lane 1, marker; lane 2, *Bp*WT; and lane 3, *Bp∆tig*. Immunoblot analysis of proteinase K-treated whole cell extracts of *Bp*WT and *Bp∆tig* using the (**D**) anti-*B. pseudomallei* LPS (α-*Bp*LPS) and (**E**) anti-*B. pseudomallei* CPS (α-*Bp*CPS). Lane 1, marker; lane 2, *Bp*WT; and lane 3, *Bp∆tig*.

When culturing the bacterial strains, it was consistently observed that, compared to the parent strain, the *Bp∆tig* mutant displayed a hyper-mucoid colony morphology type (Fig. 5A; Fig. S3) This colony type is usually associated with the dysregulation of extracellular DNA or *O*-polysaccharide modifications of the lipopolysaccharide (LPS) (60), which has been shown to have implications for the ability of the bacteria to form biofilms (61). Therefore, the biofilm-forming capacity of the *Bp∆tig* mutant was investigated. A static biofilm assay was conducted using 96-well microtiter plates as previously described (62). The *Bp∆tig* mutant demonstrated significantly increased biofilm production compared to the parent strain (Fig. 5B).

The proteomic data also revealed that the *Bp∆tig* mutant had 24 proteins belonging to the LPS and capsular polysaccharide (CPS) biosynthetic clusters that were significantly differentially present in the proteome of the *Bp∆tig* mutant (Table 1). Therefore, the potential dysregulation of the LPS and CPS was investigated through silver stain and immunoblot analysis of crude bacterial extracts. The *Bp∆tig* mutant sample had a more intense silver stain compared to the *Bp*WT, suggesting that the mutant may be producing more LPS (Fig. 5C). This was confirmed by the immunoblot analysis using anti*-B. pseudomallei* LPS, which revealed a slightly larger and stronger LPS signal in the *Bp∆tig* sample compared to the *Bp*WT sample (Fig. 5D). Immunoblot analysis of both samples for capsule expression using anti-*B. pseudomallei* CPS demonstrated that *Bp∆tig* also produced more capsule, which has a small shift in migration compared to *Bp*WT (Fig. 5E).

These data suggest that the deletion of *tig* results in a hyper-mucoid colony type and increased biofilm-formation capability, which may be due to the dysregulation of LPS and CPS biosynthetic clusters.

### Recombinant *Bp*TF harbors PPIase and chaperone-like activity

**Fig 6:**
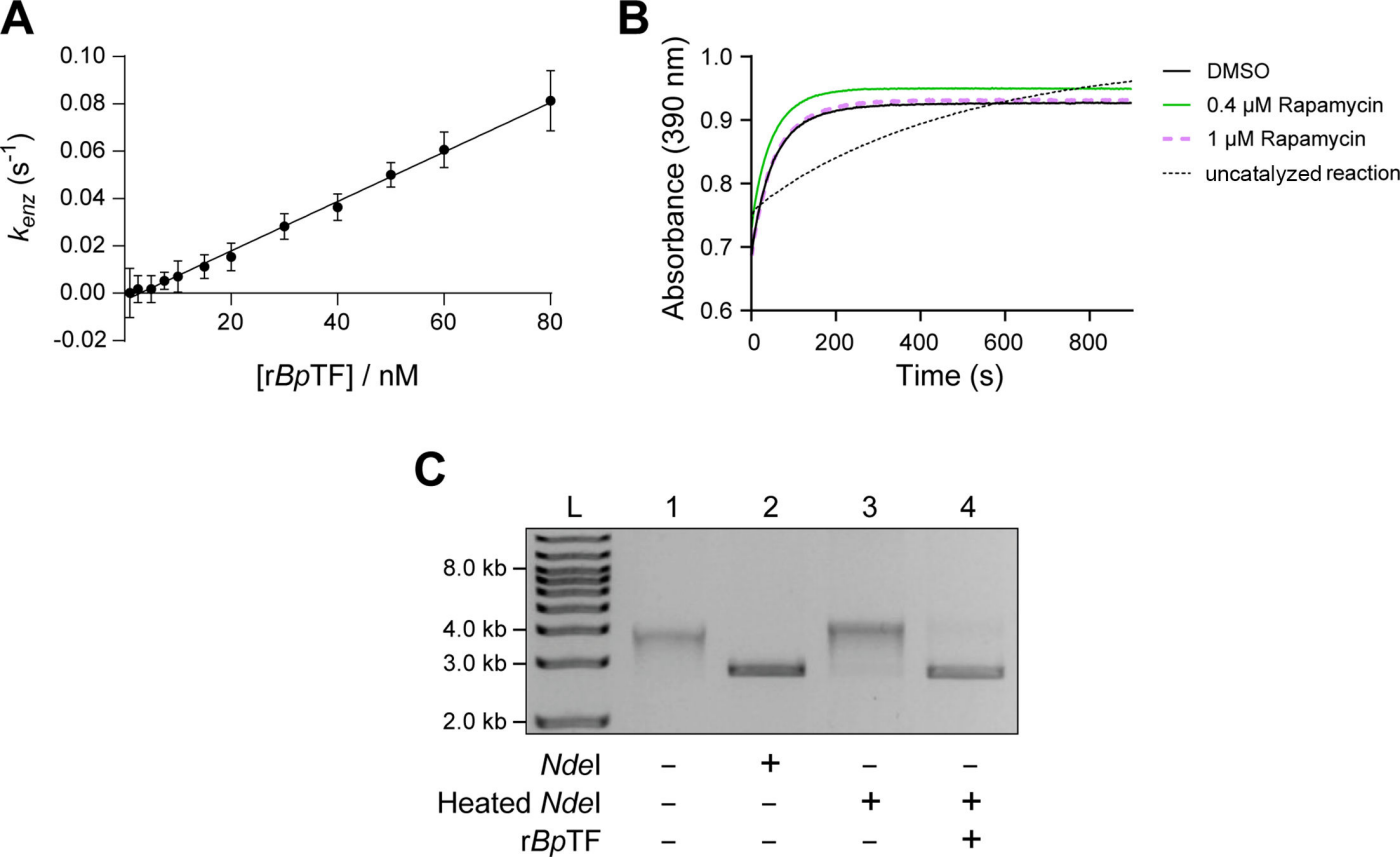
The PPIase and chaperone-like activity of the recombinantly expressed *B. pseudomallei* Trigger Factor protein. (**A**) Data for determining *k*_cat_*/K*_M_ value of *Bp*TF. Recombinant *Bp*TF has a *k*_cat_*/K*_M_ value of 1.05 ± 0.04 × 10^6^ M^−1^ s^−1^ (fit equation: *y* = 0.001045*x* − 0.003014; *R^2^* = 0.95). The rate of *Bp*TF was tested at a range of concentrations in triplicate. Error bars show standard deviation. (**B**) r*Bp*TF was incubated without (black line) or with rapamycin at a concentration of 0.4 µM (green line) or 1 µM (purple line). An uncatalyzed reaction was run as a comparator (dotted back line). r*Bp*TF was used at a concentration of 30 nM in a protease-coupled PPIase assay. (**C**) r*Bp*TF exhibits chaperone-like activity, preventing the thermal inactivation of *Nde*I. *Nde*I (10 U) was incubated with or without 30 nM r*Bp*TF at 60°C for 20 min. The mixture was cooled and then used to digest 100 ng of pUC19 for 2 h at 37°C. L, ladder; lane 1, uncut pUC19 (circular pUC19, negative control); lane 2, pUC19 incubated with active *Nde*I (linearized pUC19, positive control); lane 3, pUC19 incubated with heat-treated *Nde*I; and lane 4, pUC19 incubated with *Nde*I heat-treated in the presence of 30 nM r*Bp*TF.

To confirm PPIase and chaperone activity, recombinant *B. pseudomallei* Trigger Factor protein (r*Bp*TF) was expressed and purified. The PPIase activity of r*Bp*TF was determined using the protease-coupled PPIase assay as described previously using the substrate succinyl-Ala-Phe-Pro-Phe-*p*-nitroanilide (SAPPP) (63). In this assay, an active PPIase increases the *cis*-to*-trans* isomerization of SAPPP, and α-chymotrypsin cleaves the *trans* form, releasing *p*-nitroanilide (pNA), which can be measured at 390 nm. The *k*_cat_*/K*_M_ value for r*Bp*TF was determined to be 1.05 ± 0.04 × 10^6^ M^−1^ s^−1^ (Fig. 6A). FK506 and rapamycin are cognate inhibitors of FKBPs with potencies in the low nanomolar range. However, they are not considered to be good inhibitors of *E. coli* TF (64, 65). To test if this was also the case for *Bp*TF, rapamycin was tested against r*Bp*TF at 400 nM and 1.0 µM. Rapamycin was found not to inhibit r*Bp*TF PPIase activity at these concentrations (Fig. 6B). A multiple sequence alignment of *B. pseudomallei* Mip (*Bp*Mip) and *Bp*TF against human FK506 Binding Protein 12 (hFKBP12) showed that all of the amino acids that are considered important for macrolide binding (64, 65) are conserved in *Bp*Mip, while only 4 out of 10 are conserved in *Bp*TF, which supports why rapamycin is a poor inhibitor of r*Bp*TF (Fig. S4).

Next, the chaperone activity of r*Bp*TF was investigated using a chaperone assay described by Sharma et al*.* (66). The assay tests the ability of a chaperone protein to prevent the denaturation of the substrate protein under thermal stress. In this assay, the restriction enzyme *Nde*I was used as the model substrate protein, and its ability to digest the test plasmid pUC19 was assessed. Following incubation at 60°C for 20 min, *Nde*I in the absence of r*Bp*TF was completely inactivated and unable to digest pUC19, as the product of the reaction ran similarly to the negative control of uncut pUC19 (Fig. 6B, lanes 1 and 3). Co-incubation of *Nde*I with r*Bp*TF prevented thermal inactivation, as the product of the reaction (Fig. 6B, lane 4) ran similarly to the positive control, linearized pUC19 plasmid produced by active *Nde*I (Fig. 6B, lane 2). Together, these results demonstrate that *Bp*TF is an active PPIase, which is not inhibited by rapamycin, and that *Bp*TF can also act as a chaperone.

## DISCUSSION

The molecular structure and function of the Trigger Factor protein has been studied in detail due to its status as the primary ribosome-associated chaperone in bacteria and its prominent role in protein folding and protein complex assembly (42, 43). Despite its central role as a chaperone, the loss of TF is not lethal. In *E. coli*, the deletion of *tig* fails to produce an obvious growth phenotype or detectable levels of protein aggregation (48, 67). Instead, the loss of TF is compensated by the enhanced activity of other chaperones, such as DnaK, GroEL, and SecB, revealing a built-in redundancy in the protein folding process, where the action of several chaperones can, to varying extents, substitute for the function of another (43). Therefore, although not surprising due to its association with the ribosome, it is interesting to observe that the loss of TF in *B. pseudomallei* has such pleiotropic effects on bacterial virulence. Several studies have reported that the loss of TF has an impact on bacterial virulence (45–47). However, these have predominantly focused on gram-positive bacteria, and information about the role of TF in the virulence of gram-negative bacteria remains limited.

**Fig 7:**
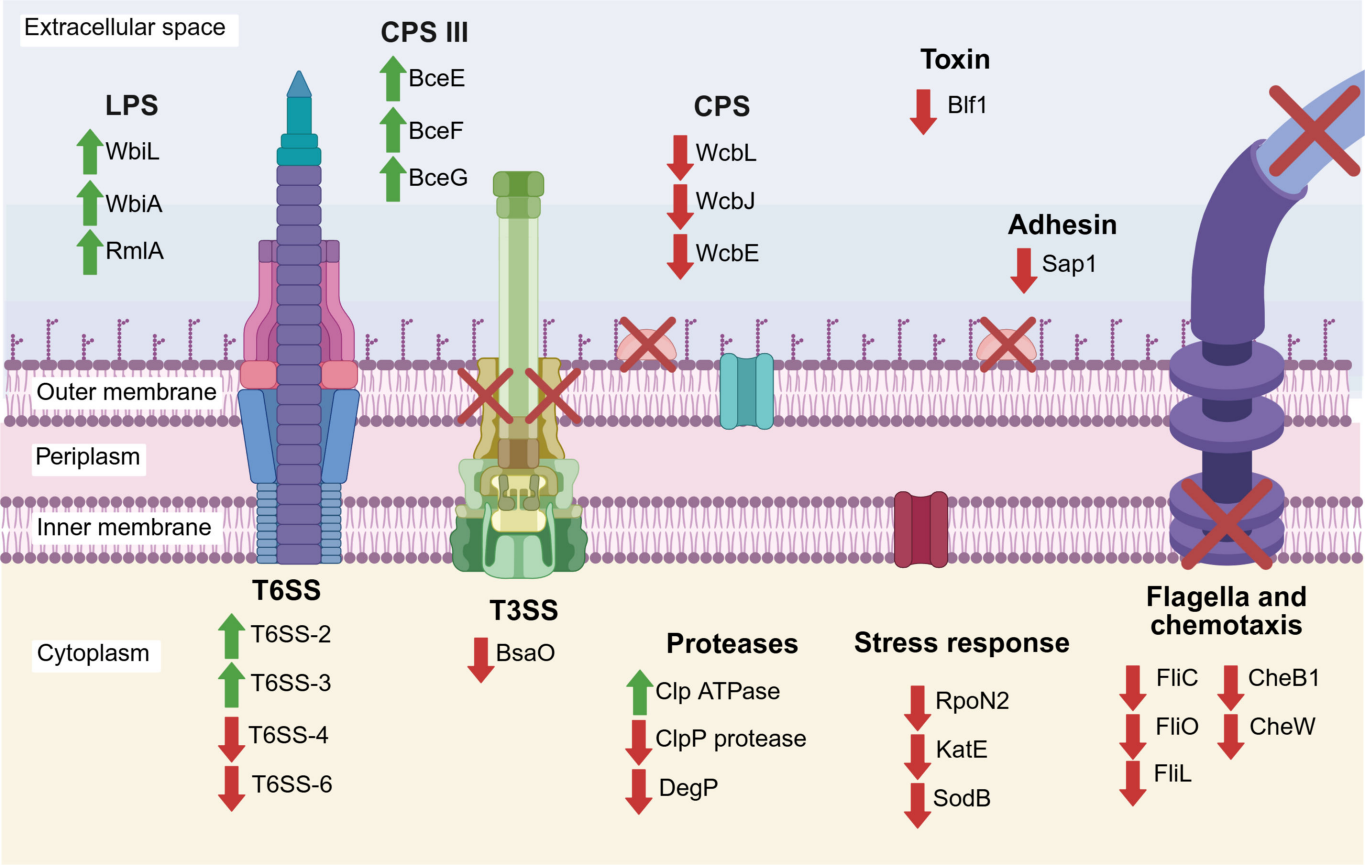
The proposed role of *Bp*TF in the virulence of *B. pseudomallei* based on phenotypic characterization and comparative proteomics analysis. BpTF may interact with virulence-associated proteins either by mediating *de novo* protein folding or by chaperoning native-like proteins and facilitating their assembly into protein complexes. Arrows are used to summarize the proteomic data of the *Bp∆tig* mutant strain, showing an increase (green upward arrow) or a decrease (red downward arrow) in protein abundance relative to the wild-type strain. Red crosses indicate putative loss of function. Loss of *Bp*TF results in the dysregulation of LPS-, EPS-, and CPS-related proteins and decreased motility via proteins that form part of the flagella and chemotaxis machinery. Furthermore, the absence of *Bp*TF leads to a decrease in stress response proteins, induction of two tightly regulated T6SS, and altered expression of quality control proteases. *Bp*TF is important for entry into host cells via Sap1 and potentially also affects the function of T3SS, as well as toxin production. Figure made in BioRender.

This study constructed and characterized a *Bp∆tig* mutant to investigate the role of *Bp*TF in *B. pseudomallei* virulence. The mutant was found to be strongly attenuated *in vitro,* and the combination of proteomic analysis coupled with phenotypic characterization revealed that several key virulence mechanisms were dramatically affected by the loss of *Bp*TF (summarized in Fig. 7).

The most striking *Bp∆tig* phenotype was the marked attenuation in the cell infection assays. First, the mutant had a significant defect in the rate of internalization, entering RAW264.7 macrophage cells at more than two orders of magnitude lower than the parent strain. This was interesting as the *tig* mutant in *B. subtilis* did not demonstrate any attenuation in entry and intracellular survival in macrophage cell lines (44). Furthermore, as demonstrated using non-phagocytosing A549 epithelial cells, the *Bp∆tig* mutant had both a reduced ability to adhere to cells and a reduced ability to enter cells after the adhesion step. A similar observation was reported for *Streptococcus suis* serotype 2, where the deletion of *tig* resulted in a significant decrease in the adherence of the bacteria to Hep-2 epithelial cells (46). Interestingly, the reduced entry of *Bp∆tig* was much more pronounced than that of the *S. suis tig* mutant. Comparative proteomics analysis identified that the *Bp∆tig* mutant proteome had 7.5 times less surface attachment protein 1 (Sap1, BPSL0097) compared to the parent strain. Sap1 is found on the outer surface of *B. pseudomallei* and has been shown to be important for mediating extended contact of *B. pseudomallei* to host cells and facilitating bacterial uptake (21). In a study by Heacock-Kang et al*.* (21), only 5% of the *B. pseudomallei* 1026b *sap1* mutant attached to host cells compared to the wild type. Compared to the defect observed with the *sap1* mutant, the *Bp∆tig* mutant demonstrated a larger defect in entry, suggesting that additional proteins contributed to the defect in host entry. In *B. pseudomallei* 1026b, a *fliC* transposon mutant showed a 90% reduction in bacterial uptake into RAW264.7 macrophage cells compared to the wild-type strain (17), demonstrating that motility is also important for host cell invasion. FliC was present at 165 times lower abundance in the *Bp∆tig* mutant proteome compared to the parent strain and represented the second most negative fold change in protein abundance following only *Bp*TF itself. In addition, flagellar proteins FliL and FliO, reported to be involved in motility in *Salmonella enterica* (68, 69), and chemotaxis proteins CheW and CheB1 (70, 71), were also significantly less abundant in the *Bp∆tig* mutant proteome. Multiple structural and effector proteins of the T3SS have been reported to be required for adhesion and invasion of *B. pseudomallei* into host cells, including BsaQ (72), BipBCD proteins (73–75), and BopE (76), to name a few. Proteomic analysis of the *Bp∆tig* mutant revealed that the outer membrane ring/secretin protein BsaO was decreased in relative abundance. Although no comprehensive study has been done to characterize BsaO in *B. pseudomallei*, a transposon library screen identified it as necessary for establishing respiratory melioidosis (51). Additionally, the deletion of BsaO homologs InvG and MxiD in *Salmonella typhimurium* and *Shigella flexneri,* respectively, led to impaired invasion of epithelial cells and diminished effector protein secretion (77, 78). Thus, it can be hypothesized that the reduced abundance of BsaO may affect proper T3SS assembly and secretion of effector proteins, thereby contributing to the observed adhesion and invasion defects of the *Bp∆tig* mutant. Further investigation, including quantification of effectors secreted by *Bp∆tig,* would confirm this hypothesis. Altogether, the results suggest that the observed defect in host cell entry by the *Bp∆tig* mutant is due to alterations in multiple virulence factors, including the reduction of the bacterial adhesins and motility, as well as the potential dysregulation of the T3SS (Fig. 7).

The next striking *Bp∆tig* mutant phenotype was the inability to effectively spread from cell to cell and produce characteristic features of *B. pseudomallei* infection, namely the formation of MNGCs and induction of cell cytotoxicity. Instead, the *Bp∆tig* mutant was able to persist inside host cells for much longer than the parent strain. A defect in MNGC formation has been attributed to the dysregulation of the Type VI secretion system (31). T6SS-1 has been shown to be essential in the formation of MNGCs in *B. pseudomallei*. Deletion of *hcp1* or *vgrG* in *B. pseudomallei* resulted in the inability to form MNGCs up to 18 h post-infection (31, 79). Although the *Bp∆tig* mutant was impaired in forming MNGCs, comparative proteomics analysis did not identify any difference in the abundance of T6SS-1 proteins. However, this is not surprising, as expression of T6SS-1 is only activated when the bacteria sense low-molecular-weight thiols in the host cytosol during infection (80). Therefore, the conditions used to generate samples for proteomic analysis may have masked any potential interaction of *Bp*TF with T6SS-1 components. However, components of other T6SS clusters were identified as differentially expressed in the *Bp∆tig* mutant, and since T6SS are known to be tightly regulated in *B. pseudomallei* (81), it is not unreasonable to hypothesize that the T6SS-1 system may also be differentially expressed *in vitro*, resulting in a decrease in MNGC formation. A similar phenotype was observed with the loss of the PPIase PpiB in *B. pseudomallei,* where the deletion of *ppiB* resulted in a 67.4% reduction in nuclei associated with MNGC formation (33). This defect was attributed to either the improper folding of T6SS cluster components or the dysregulation of T6SS regulators. It may be the same case for the *Bp∆tig* mutant. Further studies investigating protein-protein interaction of *Bp*TF with protein regulators or components of the various T6SS will further identify the role of *Bp*TF in the formation of MNGC by *B. pseudomallei.*

The intracellular counts and immunofluorescence images revealed that the host cell monolayer infected with the *Bp∆tig* mutant remained intact, and there was limited evidence of cell toxicity. This was confirmed by the lower levels of LDH released in the *Bp∆tig*-infected monolayer. Several mechanisms have been linked to *B. pseudomallei*-induced cell toxicity, including the T6SS, the production of secondary metabolites, and toxins (31, 52, 82, 83). One protein that stood out in the proteomic analysis was the reduced abundance of the Burkholderia lethal factor 1 (Blf1) in the *Bp∆tig* mutant. Blf1 has been reported to inhibit protein synthesis in host cells by deaminating the Gln339 of eukaryotic initiation factor 4 A (eIF4A), leading to host cell death (53), and the *B. pseudomallei* K96243 *blf1* mutant has been reported to be highly attenuated in mice (52). Therefore, the reduced abundance of Blf1 as well as the limited cell-to-cell spread may be the main contributors to the reduced cytotoxicity of the *Bp∆tig* mutant.

The *Bp∆tig* mutant was not able to achieve the same peak intracellular titers as the wild-type strain. This may, in part, be due to an impaired stress response, as phagocytic cells use oxidative stress via reactive oxygen and nitrogen species as well as acidification of phagosomes to kill invading pathogens (84). The *Bp∆tig* mutant was found to be more susceptible to stress induced by low pH, H_2_O_2_, and heat. Although the role of *tig* in stress response has been documented in several gram-positive pathogens, the comparative proteomics analysis conducted in this study has provided insight into possible mechanisms behind the increased sensitivity of the *Bp∆tig* mutant to these various stresses. At first glance, classical oxidative stress response enzymes responsible for detoxifying reactive oxygen species (ROS), such as superoxide dismutase protein SodB and catalase protein KatE (85), as well as its positive regulator sigma factor RpoN2 (23), were identified as significantly reduced in abundance in the *Bp∆tig* mutant. Of further interest was that several proteins belonging to T6SS-2 and T6SS-3 were significantly increased in relative abundance in the *Bp∆tig* mutant. As noted earlier, the expression of the T6SS is tightly regulated in *B. pseudomallei*. The T6SS-2 gene cluster is highly conserved in the *B. pseudomallei* complex (86), and elegant studies in *Burkholderia thailandensis* have demonstrated that T6SS-2 exports metal chelators TseM and TseZ to acquire the transition metals, Mn^2+^ and Zn^2+^, respectively, to combat ROS (87, 88). Although the functional role of T6SS-3 in *B. pseudomallei* has yet to be characterized, it has been speculated that it also plays a role in resistance to oxidative stress as T6SS-3 gene cluster expression is induced by antibiotics that promote the formation of ROS (86). Therefore, the dysregulated T6SS-2 and T6SS-3, through the deletion of *tig*, may contribute to the enhanced susceptibility of *Bp∆tig* to oxidative stress.

The role of *tig* in biofilm formation has been described previously. In *Streptococcus mutans*, the deletion of the *tig* homolog, *ropA,* led to media-dependent dysregulation in biofilm production (47), while a study in *S. aureus* reported that *tig* deletion resulted in impaired biofilm formation (45). This study has further utilized the comparative proteomics results to reveal that the biofilm defect of the *Bp∆tig* mutant may, in part, be attributed to the altered LPS and CPS production in *Bp∆tig* but is likely multi-factorial, brought about by the dysregulated production of several outer membrane components.

This study demonstrated that *Bp*TF has chaperone-like activity and has the capacity to protect proteins from thermal denaturation. Therefore, it would not be surprising that the deletion of *tig* resulted in an increased susceptibility of the *Bp∆tig* mutant to heat stress. A *S. suis tig* mutant was similarly more susceptible to thermal stress at 41°C (46). Interestingly, the opposite was observed in *E. coli* and *L. monocytogenes* studies, which demonstrated that the absence or reduction of TF led to improved survival at higher temperatures (50°C) (44, 89). Proteases are known to play a role in the stress response of bacteria through their ability to degrade stress-induced misfolded proteins (90–92). Comparative proteomic analysis of the *Bp∆tig* mutant revealed significant differences in the relative abundance of various protease components. A DegP-like protease and the ClpP protease subunit of the Clp chaperone-proteases were identified as reduced in relative abundance. DegP functions as both a chaperone and a protease and has been shown to play an important role in mediating acidic, oxidative, and heat resistance of pathogens such as *E. coli* and *Streptococcus pyogenes* (90, 93, 94). Clp proteases are the most prevalent energy-dependent proteases in bacteria and have been shown to play a role in the stress response through the degradation of aggregated proteins (95, 96). Altogether, the results reveal that multiple stress resistance pathways are affected by the loss of *Bp*TF, and the increase in numerous T6SS components suggests that the *Bp∆tig* mutant is either under stress in enriched growing environments or has become dysregulated as a result of the malfunction of their many regulators.

In line with studies on *E. coli* TF (64, 65), rapamycin was found not to inhibit r*Bp*TF even at 300 times the *K_i_* for *Bp*Mip (32). It has been hypothesized that the decrease in inhibition potency of FK506 and rapamycin against TFs is a result of differences in several key macrolide-binding residues that are highly conserved in other FKBP family members (64, 65). In the age of artificial intelligence and enhanced molecular modeling and structure-based drug design methods, it should be possible to identify potential inhibitors against TF or *Bp*TF specifically. Such computational modeling approaches have recently been used to identify potential inhibitors of *Mycobacterium tuberculosis* TF (97). It would be interesting to test these or similarly identified inhibitors against *Bp*TF and *B. pseudomallei* to ascertain if *Bp*TF can be developed into a therapeutic target to improve the treatment of melioidosis.

In conclusion, this study showed the association of the FKBP *Bp*TF with virulence in *B. pseudomallei.* Characterization of the *Bp∆tig* mutant revealed that the loss of *Bp*TF affects several virulence determinants of *B. pseudomallei,* including motility, stress resistance, adhesion and invasion, polysaccharide biosynthesis, and biofilm formation, all culminating in the attenuation of the mutant strain *in vitro*. Further studies will be required to validate the proteomic results and confirm the protein interaction partners of *Bp*TF, but the data presented here indicate that *tig* is essential for *B. pseudomallei* virulence and warrant further investigation as a target for the development of new therapies to combat this multi-drug-resistant pathogen.

## MATERIALS AND METHODS

### Bacterial strains and growing conditions

The bacterial strains and plasmids used in the study are listed in Table 2. Bacterial strains were grown in Luria-Bertani broth overnight at 37°C with agitation unless otherwise stated. Antibiotics were added at the following final concentrations: chloramphenicol at 30 µg/mL, ampicillin at 100 µg/mL, while the inducer isopropyl β-D-1-thiogalactopyranoside (IPTG) was added at a final concentration of 1 mM.

**TABLE 2:** Bacterial strains and plasmids used in this study

Plasmid/strain	Description	Source or reference
Plasmids		
pDM4	Suicide vector harboring the *sacB* gene, Cm^R^	(98)
pDM4_*tig_*del	Derivative of pDM4, containing the *tig* deletion construct, Cm^R^	This study
pET-15b	Broad host range vector for expression, Amp^R^	Novagen
pET-15b_*tig*	Derivative of pET-15b, encoding the *tig* gene, Amp^R^	Dr. Isobel Norville, DSTL
pUC19	Plasmid substrate used for the chaperone assay, Amp^R^	Invitrogen
*Escherichia coli*		
DH5α	F^−^ ɸ80*lacZ*ΔM15 *endA1 recA1 hsdR17*(r_K_^−^ m_K_^+^) *phoA sup*E44 *thi-1* Δ*gyr*A96 *(ΔlacZYA-argF)U169 relA1F* λ*^−^*	Invitrogen
S17-1 λpir	*recA thi pro hsdR^−^ M^+^* RP4::2-Tc::Mu::Km Tn7 Tp^R^ Sm^R^ λ*pir. E. coli* S17-1 strain with a prophage carrying the *pir* gene used for conjugating pDM4 derivatives into *B. pseudomallei*	(99)
BL21 (DE3) pLysS	*E. coli* expression strain	Invitrogen
*Burkholderia pseudomallei*		
K96243 (*Bp*WT)	Clinical isolate	DSTL (54)
*Bp∆tig*	K96243 derivative; unmarked deletion *Δtig*	This study

### Construction of the *Bp∆tig* deletion mutant

Construction of an in-frame deletion mutant was performed using the mutagenesis protocol as described by Logue et al*.* (50). In brief, the upstream and downstream DNA regions flanking the *BPSL1402 (tig)* gene were amplified by PCR with Phusion polymerase using *B. pseudomallei* K96243 genomic DNA as a template and the primer pairs *Bp*Tig_UP_FWD and *Bp*Tig_UP_REV, and *Bp*Tig_DOWN_FWD and *Bp*Tig_DOWN_REV, respectively (Table 3). The primers were used to introduce the restriction site for *Bgl*II to facilitate ligation of the two flanks to form the *tig_*del deletion construct and *Xba*I to ligate the deletion construct into the pDM4 plasmid to generate the final suicide plasmid pDM4_*tig_*del, which was confirmed by sequencing (Australian Genome Research Facility). The suicide plasmid was transformed into *E. coli* S17-λpir, and this strain was used to conjugate pDM4_*tig_*del into *B. pseudomallei* strain K96243 (*Bp*WT). Merodiploid clones were selected via double selection on LBA containing ampicillin and chloramphenicol and confirmed by PCR of purified genomic DNA. Counterselection based on *sacB* was used to select for excision of the pDM4 backbone by plating the merodiploid clone onto LB agar lacking sodium chloride and supplemented with 10% sucrose. Chloramphenicol-sensitive colonies were screened by PCR to confirm the loss of *tig* and the generation of the desired *Bp∆tig* mutant strain. The *Bp∆tig* mutant was sequenced using a PacBio sequencing platform at Genomics WA. The whole-genome sequencing data were aligned to the K96243 reference genome (NC_006350.1 for chromosome 1 and NC_006351.1 for chromosome 2).

**TABLE 3:** Primers used in this study

Primer	Sequence^*a*^	Use
*Bp*Tig_Up_FWD	TCTAGAGATCCCTTGAACGAATCGGGTGC	Amplification of the upstream flanking region of *tig*
*Bp*Tig_Up_REV	AGATCTCAATCGTCCTAAAATTATTCG	
*Bp*Tig_Down_FWD	AGATCTAATACTCATTCTCGGACAAGGC	Amplification of the downstream flanking region of *tig*
*Bp*Tig_Down_REV	TCTAGAGCAGCGCGTAGCGCTTGCCCTTCG	
*Bp*Tig_Exp_FWD	CATATGGCTAACGTTGTTGAA	Amplification of *tig* for protein expression and purification
*Bp*Tig_Exp_REV	GGATCCTTATGCTTGCGCCGTCGC	

^*a*^ Restriction sites are underlined.

### Growth curves

To investigate if deletion of *tig* affected *B. pseudomallei* fitness, growth curves were performed as follows. Overnight cultures of *Bp*WT and *Bp∆tig* were adjusted to 4 × 10^8^ CFU per mL in LB broth. This standardized inoculum was diluted 1:50 into 20 mL of fresh LB broth in a 50 mL conical tube and incubated at 37°C with agitation. At the indicated time points, the colony-forming units were enumerated by removing a 200 µL sample and plating out appropriate serial dilutions onto LB agar plates.

### Cell-based infection assays

RAW264.7 murine macrophage and A549 epithelial cells were routinely cultured in Dulbecco’s modified Eagle’s medium (Gibco, Life Technologies) supplemented with 1% (vol/vol) GlutaMAX (Gibco, Life Technologies), 10% (vol/vol) heat-inactivated Fetal Bovine Serum (FBS) (Gibco, Life Technologies), and Penicillin-Streptomycin (1,000 U/mL) (Gibco, Life Technologies) and grown at 37°C in 5% CO_2_. For infection assays, the culture medium was changed to Leibovitz L-15 medium (L-15) (Gibco, Life Technologies) supplemented with 1% (vol/vol) GlutaMAX and 10% (vol/vol) FBS, and cultures were incubated at 37°C under normal atmosphere.

Cells were seeded into 24-well plates in 1 mL volumes at 4 × 10^5^ cells per well and incubated for 20 h to achieve a confluent monolayer of approximately 1 × 10^6^ cells per well prior to infection. Overnight cultures of *Bp*WT and *Bp∆tig* were adjusted to an absorbance of 0.4 and 0.7 at 580 nm, respectively, in L-15 to achieve approximately 4 × 10^8^ CFU/mL. The standardized inocula were diluted 1:10 in L-15, and 1 mL aliquots were added to each well to achieve a multiplicity of infection of 10. Cells were infected for 30 min or 1 h for RAW264.7 and A549 cell infection assays, respectively. Following infection, bacteria were aspirated, and the infected monolayer was washed three times with 1 mL PBS. The infected cells were then incubated with L-15 containing 1 mg/mL of kanamycin for 30 min to kill the remaining extracellular bacteria. The medium was then replaced with L-15 containing 0.25 mg/mL kanamycin (deemed time 0 h post-infection) and incubated for up to 48 h. At the indicated time points, the medium was replaced with 1 mL of MilliQ water to lyse the cells and release the intracellular bacteria. The cell lysate was then serially diluted in PBS and plated out onto LB agar plates to enumerate the number of bacteria.

The percentage of bacterial internalization was calculated as the number of bacteria recovered after incubation of cells with L-15 containing 1 mg/mL of kanamycin (time 0 h post-infection) divided by the number of bacteria in the infection inoculum multiplied by 100.

### Adhesion to A549 cells

A549 lung epithelial cells were prepared and infected with the bacterial strains as described above. After 1 h of infection, the monolayer was washed three times with 1 mL of PBS to remove nonadherent cells. Cells were then lysed with 1 mL of MilliQ, and the resulting suspension was serially diluted in PBS and plated out onto LB agar for bacterial enumeration. The percentage of bacterial adhesion was calculated as the number of bacteria recovered after infection divided by the number of bacteria in the infection inoculum multiplied by 100.

### *B. pseudomallei* cytotoxicity assay

The toxicity of the *B. pseudomallei* strains was assessed by measuring the lactate dehydrogenase in the cell culture supernatant at 24 h pi using the Roche Cytotoxicity Detection Kit (Roche, Merck) as per the manufacturer’s instructions. To express the results as a percentage of *Bp*WT cytotoxicity, the LDH released in the *Bp∆tig*-infected monolayer was divided by the LDH released in the *Bp*WT-infected monolayer, multiplied by 100.

### Immunofluorescence

RAW 264.7 murine macrophages were seeded onto 13 mm round coverslips in 24-well plates at a concentration of 4 × 10^5^ cells per well and incubated for 20 h. The macrophage cells were infected as described above for cell-based infection assays. At the indicated time points, the medium was removed, and the cell monolayers were washed three times with PBS for 5 min each. The monolayers were then fixed with 4% (wt/vol) PFA for 1 h at room temperature and then washed again with PBS, three times for 5 min each. Monolayers were stained at room temperature as follows. Samples were first permeabilized with 0.1% (vol/vol) Triton X-100 in PBS for 15 min and then blocked with 5% (vol/vol) FBS in PBS (blocking buffer) for 2 h. Samples were then incubated with the primary antibody anti-*B. pseudomallei* LPS diluted to 1 µg/mL (1:100; α-*Bp*LPS, clone Mab4VIHXII) in blocking buffer for 1 h. Samples were washed in PBS and then incubated with secondary antibody Anti-mouse whole IgG-fluorescein isothiocyanate (1:64; Sigma-Aldrich) and F-actin stain Alexa Fluor 647-labeled Phalloidin (1:1,000; ThermoFisher Scientific) in blocking buffer for 1 h. Samples were washed again, and nuclei were stained for 15 min using Hoechst 33258 (1:10,000; ThermoFisher Scientific). Following final washes with PBS, coverslips were mounted onto glass slides using Prolong Gold Antifade Mountant (Molecular Probes). Fluorescence microscopy was performed using a Nikon A1Si confocal microscope, and images were acquired using NIS-Elements software (Nikon).

### MNGC enumeration

MNGC formation in *Bp*-infected monolayers was evaluated as described previously (100). In brief, 1,000 nuclei were counted, and the percentage of MNGCs formed was calculated as the number of nuclei associated with MNGCs divided by the number of nuclei counted, multiplied by 100.

### Motility assay

The motility of the *B. pseudomallei* strains was assessed as described previously (33). In brief, overnight cultures were prepared, and a small aliquot was inoculated into the center of a 0.3% LB motility agar plate using a sterile 1 µL inoculation loop. Plates were incubated upright at 37°C, and the diameter of bacterial spread was measured at 24 h post-inoculation.

### Stress assays

To investigate if *tig* was required for *B. pseudomallei* stress resistance, overnight cultures were prepared and used in the following tests.

#### Oxidative stress

Overnight cultures were diluted 1:100 into fresh LB broth and grown for 2 h to mid-log phase. Aliquots of the mid-log cultures were transferred into a 24-well plate, and an equal volume of freshly prepared H_2_O_2_-adjusted LB broth was added to obtain a final assay volume of 1 mL containing 1 mM H_2_O_2_. Bacteria were incubated at 37°C for 1 h with agitation, and CFU counts were then enumerated by plating out appropriate serial dilutions onto LB agar plates. Percentage survival was calculated as the ratio of CFU/mL in the sample with H_2_O_2_ exposure to the CFU/mL in the sample without H_2_O_2_, multiplied by 100.

#### Acidic stress

Overnight cultures were diluted and grown to mid-log phase as above. Aliquots of the mid-log cultures were diluted 1:100 into 10 mL of citrate-phosphate buffer (100 mM citric acid and 200 mM Na_2_HPO_4_, pH 4.0). Bacteria were incubated at 37°C for 1 h with agitation, and CFU counts were then enumerated by plating out appropriate serial dilutions onto LB agar plates. Percentage survival was calculated as the ratio of CFU/mL at each time point over the CFU/mL in the sample at 0 h of exposure, multiplied by 100.

#### Heat stress

The heat stress assay was conducted using methods previously described (101) with minor modifications. In brief, overnight cultures of *B. pseudomallei* were standardized to an absorbance of 0.17 at 580 nm, and a 1 mL aliquot of the adjusted bacterial culture was placed in a pre-warmed tube and incubated at 50°C. The heated cultures were sampled at 15, 30, 45, and 60 min to enumerate the CFU/mL. Percentage survival was calculated as the ratio of CFU/mL at each time point over the CFU/mL in the sample pre-heat exposure, multiplied by 100.

### Biofilm assay

The biofilm formation assay was conducted as described by Moran et al*.* (62).

### Polysaccharide microextraction for silver stain and immunoblot analysis

Samples for LPS and CPS analysis were prepared as per Apicella et al. (102) with modifications. Overnight cultures of *Bp*WT and *Bp∆tig* were adjusted to an absorbance of 0.4 and 0.7 at 580 nm, respectively, in PBS to achieve approximately 4 × 10^8^ CFU/mL. A 1 mL aliquot of the standardized culture was centrifuged to pellet the bacterial cells. The supernatant was discarded, and the bacterial cell pellet was resuspended in 100 µL of LPS buffer (2% SDS, 4% β-mercaptoethanol, 0.1% bromophenol blue, 10% glycerol, and 1 M Tris-HCl [pH 6.8]) and incubated at 95°C for 1 h to kill the bacteria. After incubation, the samples were cooled to room temperature before the addition of 5 µL of Proteinase K (20 mg/mL; Invitrogen, USA) and incubated overnight at 55°C. Treated samples were run on 15% SDS-PAGE gels and visualized by silver staining or electro-transferred to a PVDF membrane.

#### Silver staining

SDS-PAGE gels were placed in 100 mL of oxidative solution (0.7% [wt/vol] periodic acid, 40% [vol/vol] ethanol, and 5% [vol/vol] acetic acid in MilliQ water) for 10 min before rinsing the gel with 200 mL of MilliQ water for 10 min, three times. The water was decanted, and the gel was stained with freshly made 150 mL staining solution (28 mL of 0.1 M NaOH, 2 mL concentrated ammonium hydroxide added to 115 mL of MilliQ water, followed by the careful addition of 5 mL 20% [wt/vol] silver nitrate) for 10 min. Afterward, the staining solution was removed, and the gel was washed three times with 200 mL of MilliQ water for 10 min before submerging the gel in 200 mL formaldehyde developer solution (10 mg citric acid and 100 µL of 37% formaldehyde in 200 mL MilliQ water) for 10–15 min until the LPS is visible. The reaction was stopped by soaking the gel in a 100 mL 10% (vol/vol) acetic acid stopping solution. The stained gel was imaged via a flatbed scanner.

#### LPS and CPS detection by Western blot

For the detection of LPS, mouse anti-*B. pseudomallei* lipopolysaccharide (α-*Bp*LPS, clone Mab4VIHXII) was used at a dilution of 1:1,000. For the detection of CPS, mouse anti-*B. pseudomallei* capsule (α-*Bp*CPS, clone 4VIHI21507G) was used at a dilution of 1:2,000. Secondary antibody rabbit anti-mouse-HRP (Jackson ImmunoResearch Laboratories) was used at a dilution of 1:10,000, and detection of the secondary HRP conjugate was accomplished by chemiluminescence (Sigma) using a UVITEC Alliance Q9 Advanced imager.

### Cloning, expression, and purification of *Bp*TF

The open reading frame of *BPSL1402 (tig)* was amplified by PCR with Phusion polymerase using *B. pseudomallei* strain K96243 genomic DNA as a template and primer pairs *Bp*Tig_Exp_FWD and *Bp*Tig_EXP_REV (Table 3). The amplified product was cloned into the *Nde*I and *Bam*HI sites of plasmid pET15b (Novagen) to generate the expression plasmid pET-15b_*tig*. This construct was transformed into *E. coli* BL21 (DE3) pLysS to recombinantly express *Bp*TF fused to a N-terminal hexahistidine tag, r*Bp*TF.

For the protein expression and purification, an overnight culture of *E. coli* BL21 (DE3) transformed with pET-15b_*tig* was used to inoculate 800 mL of LB broth supplemented with 100 µg/mL ampicillin, which was incubated with agitation at 37°C until an optical density at 600 nm of 0.4–0.6 was reached. Expression was then induced by the addition of 1 mM IPTG followed by incubation for 4 h. Bacterial cells were harvested and lysed, and the recombinant protein was purified by nickel affinity chromatography (HisTrap, Cytiva), followed by size exclusion chromatography (Superdex 200, Cytiva) as described by Iwasaki et al*.* (37). Purified r*Bp*TF protein was assessed as having >95% purity by SDS-PAGE. The concentration was calculated from the absorbance at 280 nm, and the theoretical extinction coefficient was determined using the ProtParam tool from ExPASy. Aliquots of purified r*Bp*TF were snap frozen in liquid nitrogen and stored at −80°C.

### Protease-coupled PPIase assay

The peptidyl prolyl *cis/trans* isomerase activity of the recombinant r*Bp*TF was determined using the protease-coupled PPIase assay as described previously (63). Briefly, varying concentrations of r*Bp*TF were incubated in 35 mM HEPES buffer (pH 7.8) with the substrate succinyl-Ala-Phe-Pro-Phe-*p*-nitroanilide (10 mg/mL; Bachem) for 6 min at 6°C. Chymotrypsin (Sigma) was added (250 μL of 5 mg/mL), and hydrolysis of the substrate was measured at 390 nm at 2 s intervals for 900 s (Shimadzu 1800 UV/Vis).

The pseudo-first-rate constant was calculated using the equation ln (*A_∞_ - A_t_*) = *−k_obs_t* + ln (*A_∞_ − A_0_*). Data from the first 10–50 s were taken, as these data were after the initial lag phase but before the substrate became limiting, and the *k*_obs_ was calculated by linear regression. The enzymatic rate was determined using the equation *k*_enz_ *= k*_obs_ *– k*_uncat_ (comparing the observed rate to the uncatalyzed rate). The specificity constant (*k*_cat_*/K*_m_) was then calculated using the equation (*k*_cat_*/K*_m_) = (*k*_enz_/[PPIase]) (103). The data were taken using 1, 2.5, 5, 7.5, 10, 15, 20, 30, 40, 50, 60, and 80 nM of enzyme and were fit using linear regression.

For the inhibition assay, rapamycin at 400 nM or 1 µM concentration was added to the cuvette after the addition of the protein (30 nM) but before the addition of the substrate SAPPP and incubated for 6 min at 6°C. Chymotrypsin was added (250 μL of 5 mg/mL), and hydrolysis of the substrate was measured at 390 nm at 2 s intervals for 900 s (Shimadzu 1800 UV/Vis).

### *Nde*I chaperone assay

The ability of r*Bp*TF to prevent the thermal inactivation of *Nde*I was assessed using the methods described by Sharma et al*.* (66) with modifications. In brief, 10 U of *Nde*I was heat inactivated at 60°C for 20 min with or without the presence of 30 nM of r*Bp*TF. This mixture was then incubated with 100 ng of pUC19 for 2 h at 37°C. The reaction mixture was then run on a 1% agarose gel containing 1× Syber Safe at 100 V for 50 min and visualized using a UVITEC Alliance Q9 Advanced imager.

### Protein clean-up and in-solution digestion

Cells grown on Blood agar were harvested in PBS, washed, and then lysed in lysis buffer (4% SDS, 10 mM dithiothreitol, and 100 mM Tris, pH 8.5) by boiling for 10 min. Bicinchoninic acid protein assay was used to assess the protein content as per the manufacturer’s instructions, and 1 mg of protein was precipitated overnight in 80% ice-cold acetone at −20°C. Acetone-precipitated proteins were collected and dried prior to being solubilized in 4% SDS and 100 mM Tris (pH 8.5) by boiling for 10 min at 95°C. Two hundred micrograms of each biological replicate was prepared for digestion using a Mini S-traps column (Protifi, USA) according to the manufacturer’s instructions. Briefly, samples were reduced with 10 mM dithiothreitol for 10 min at 95°C and then alkylated with 40 mM iodoacetamide in the dark for 1 h. Samples were acidified to 1.2% phosphoric acid and diluted with seven volumes of S-trap wash buffer (90% methanol and 100 mM tetraethylammonium bromide, pH 7.1) before being loaded onto S-traps and washed three times with 400 μL of S-trap wash buffer. Samples were then digested with 2 μg of Trypsin/Lys-C (Promega, 1:100 protease/protein ratio) in 100 mM tetraethylammonium bromide overnight at 37°C before being collected by centrifugation with washes of 100 mM tetraethylammonium bromide, followed by 0.2% formic acid, and then by 0.2% formic acid/50% acetonitrile. Samples were dried down and further cleaned up using C18 Stage (104, 105) tips to ensure the removal of any particulate matter.

### Reverse phase liquid chromatography–mass spectrometry

Prepared C18 purified peptides from each sample were re-suspended in Buffer A* (2% acetonitrile and 0.01% trifluoroacetic acid) and separated using a two-column chromatography setup composed of a PepMap100 C_18_ 20 mm by 75 μm trap and a PepMap C_18_ 500 mm by 75 μm analytical column (Thermo Fisher Scientific). Samples were concentrated onto the trap column at 5 μL/min for 5 min with Buffer A (0.1% formic acid and 2% DMSO) and then infused into a timsTOF Pro (Bruker) at 300 nL/min via the analytical columns using a Dionex Ultimate 3000 UPLCs (Thermo Fisher Scientific). One hundred twenty-minute analytical runs were performed by altering the buffer composition from 2% Buffer B (0.1% formic acid, 77.9% acetonitrile, and 2% DMSO) to 22% B over 95 min, then from 22% B to 40% B over 10 min, and then from 40% B to 80% B over 5 min. The composition was held at 80% B for 5 min and then dropped to 2% B over 2 min before being held at 2% B for another 8 min. The timsTOF Pro was operated in DDA PASEF mode using Compass Hystar 5.1.8.1 with a 1.1 s cycle time. Identification and LFQ analysis were accomplished using MaxQuant (version 1.6.17.0), with searches performed against the *B. pseudomallei* strain K96243 proteomes (UniProt accession: UP000000605). Samples were searched allowing oxidation on methionine as a variable modification and carbamidomethylation of cystine as a fixed modification. Both the LFQ and “Match Between Run” options were enabled to allow comparison between samples. The resulting data files were processed using Perseus (version 1.4.0.6) (106) to compare growth conditions using Student’s *t*-tests and Pearson correlation analyses.

### Mass spectrometry data analysis

MaxQuant (version 1.5.3.1) was used to identify the proteins. The searches were performed against the *B. pseudomallei* strain K96243 proteomes with carbamidomethylation of cysteine set as a fixed modification.

### Statistical analysis

Data were analyzed using Microsoft Excel, and statistical analysis was performed using GraphPad Prism (version 10.2.2).

## Data Availability

The mass spectrometry proteomics data have been deposited to the ProteomeXchange Consortium via the PRIDE (107) partner repository with the data set identifier PXD067556.

## SUPPLEMENTAL MATERIAL

Supplemental figures jb.00124-26-s0001.docx Figures S1 to S5.

Table S1 jb.00124-26-s0002.xlsx Proteomics data.

ASM does not own the copyrights to Supplemental Material that may be linked to, or accessed through, an article. The authors have granted ASM a non-exclusive, world-wide license to publish the Supplemental Material files. Please contact the corresponding author directly for reuse.
