# Supplementary material for: Trigger Factor in Burkholderia pseudomallei is essential for key virulence determinants, including host cell internalization, cytotoxicity, motility, and stress resistance
Source: J Bacteriol. 2026 Apr 22;208(5):e00124-26. doi: 10.1128/jb.00124-26 (PMC13192269; doi:10.1128/jb.00124-26)
Supplement: Supplemental figures — Figures S1 to S5. [file jb.00124-26-s0001.docx]

**Supplementary information**

# Trigger Factor in *Burkholderia pseudomallei* is essential for key virulence determinants including host cell internalisation, cytotoxicity, motility and stress resistance.

Justine B. Bendo^a^, Aleksandra W. Debowski^a,b^, Nicole M. Bzdyl^a,C^, Jua Iwasaki^a,c,d^, Josephine Starr^a^, Charles S. Bond^b^, Nichollas E. Scott^e^, Keith A. Stubbs^b,f^, Mitali Sarkar-Tyson^a^#

^a^Marshall Centre for Interventions in Infectious Diseases, School of Biomedical Sciences, University of Western Australia, Perth, Western Australia, Australia

^b^School of Molecular Sciences, The University of Western Australia, Crawley, Western Australia, Australia

^c^Wesfarmers Centre for Vaccines and Infectious Diseases, The Kids Research Institute, Australia, University of Western Australia, Nedlands, Western Australia, Australia

^d^Centre for Child Health Research, University of Western Australia, Perth, Western Australia, Australia

^e^Department of Microbiology and Immunology, University of Melbourne at the Peter Doherty Institute for Infection and Immunity, Parkville, Victoria, Australia

^f^ARC Training Centre for Next-Gen Technologies in Biomedical Analysis, School of Molecular Sciences, The University of Western Australia, 35 Stirling Hwy, Crawley, 6009, Australia

#Address correspondence to Dr Mitali Sarkar-Tyson, [mitali.sarkar-tyson@uwa.edu.au](mailto:mitali.sarkar-tyson@uwa.edu.au)





**FIG S1** Deletion of *BPSL1402* (*tig*) did not affect the growth of *B. pseudomallei* in LB broth**.** The growth of the parent strain *Bp*WT and the *Bp∆tig* mutant strain was assessed by standardising overnight cultures and diluting them to approximately 5 х 10^6^ CFU/mL in fresh LB. The cultures were grown at 37°C with agitation and sampled at indicated timepoints up to 24 h. The samples were serially diluted and plated out onto LB agar to enumerate the CFU/mL in each sample. The graph is the result of 3 biological replicates and the error bars represent the standard error of the mean. *p* values were determined using multiple unpaired *t*-tests with Welch correction.


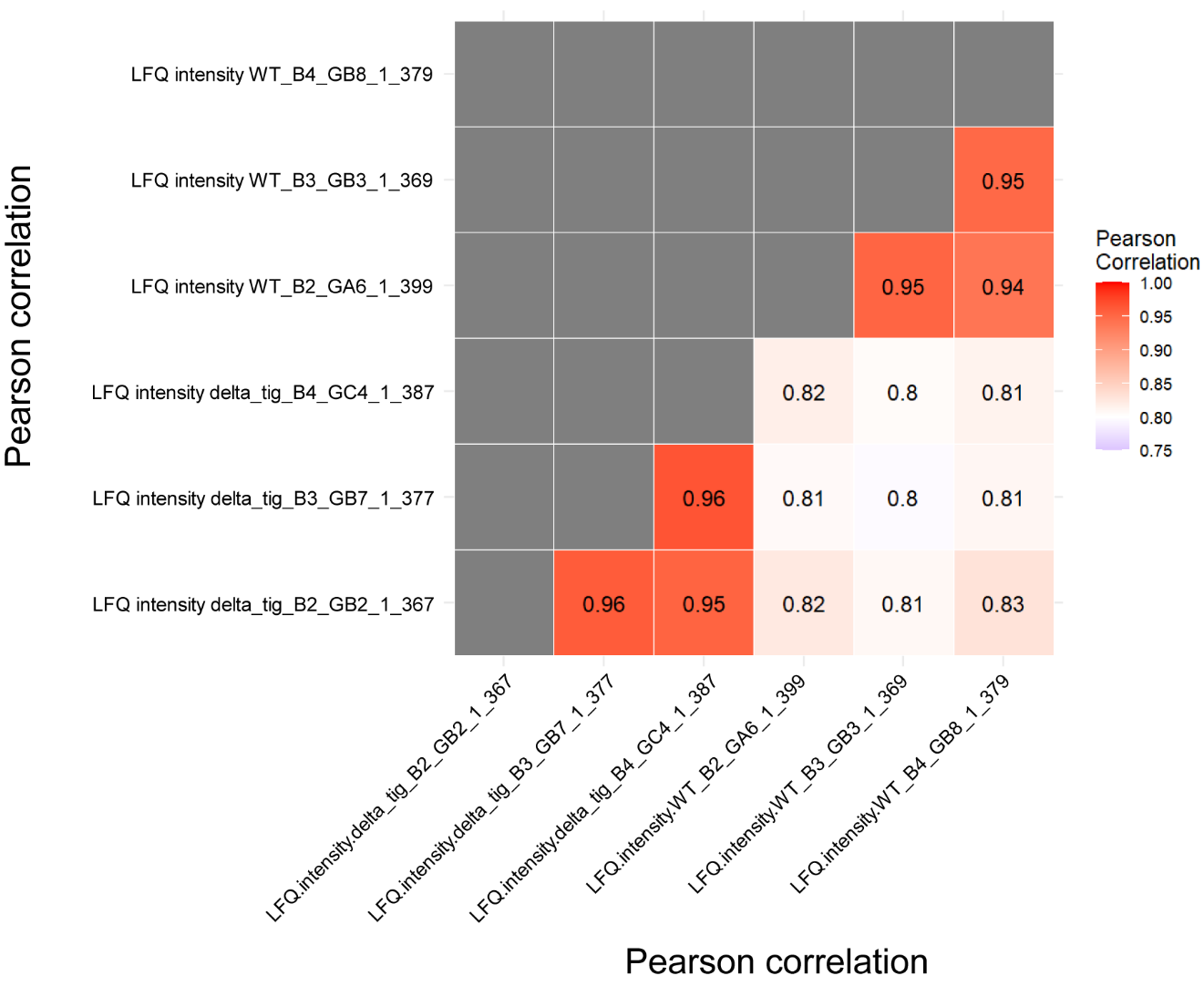


**FIG S2** Pearson correlation of the label-free-based quantitative replicates. To assess the consistency of the biological replicates for the proteomic analysis, Pearson correlation values for the unimputed LFQ values are provided. The heatmap shows each replicate with the Pearson correlation values for each pairwise comparison.


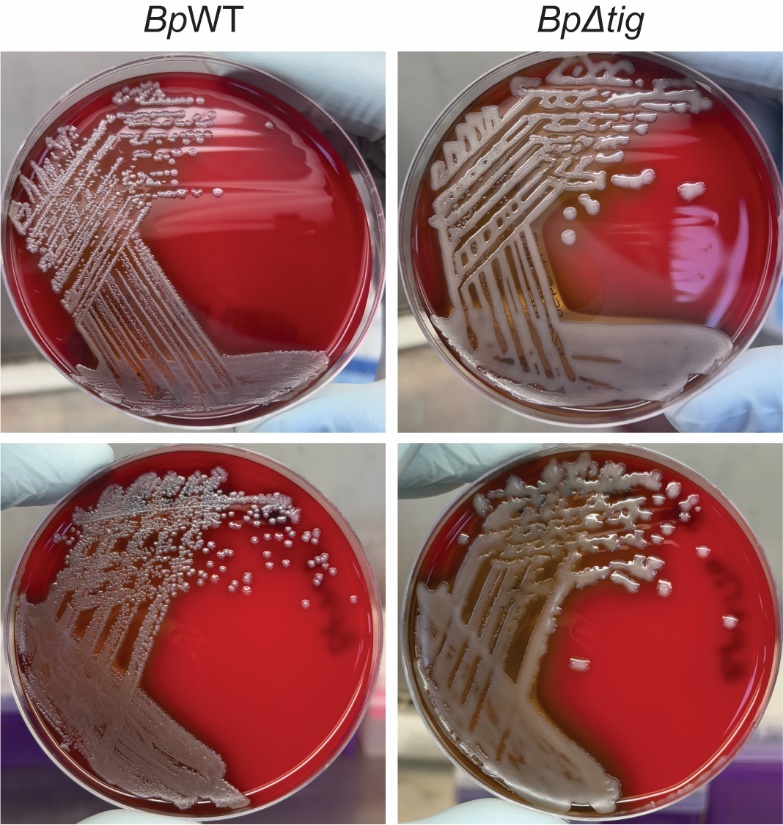


**FIG S3** The colony morphology of *Bp*WT and *Bp∆tig* mutant. The *Bp*WT parent and the *Bp∆tig* mutant strains were streaked onto CBA plates and incubated at 37°C for 48 h.


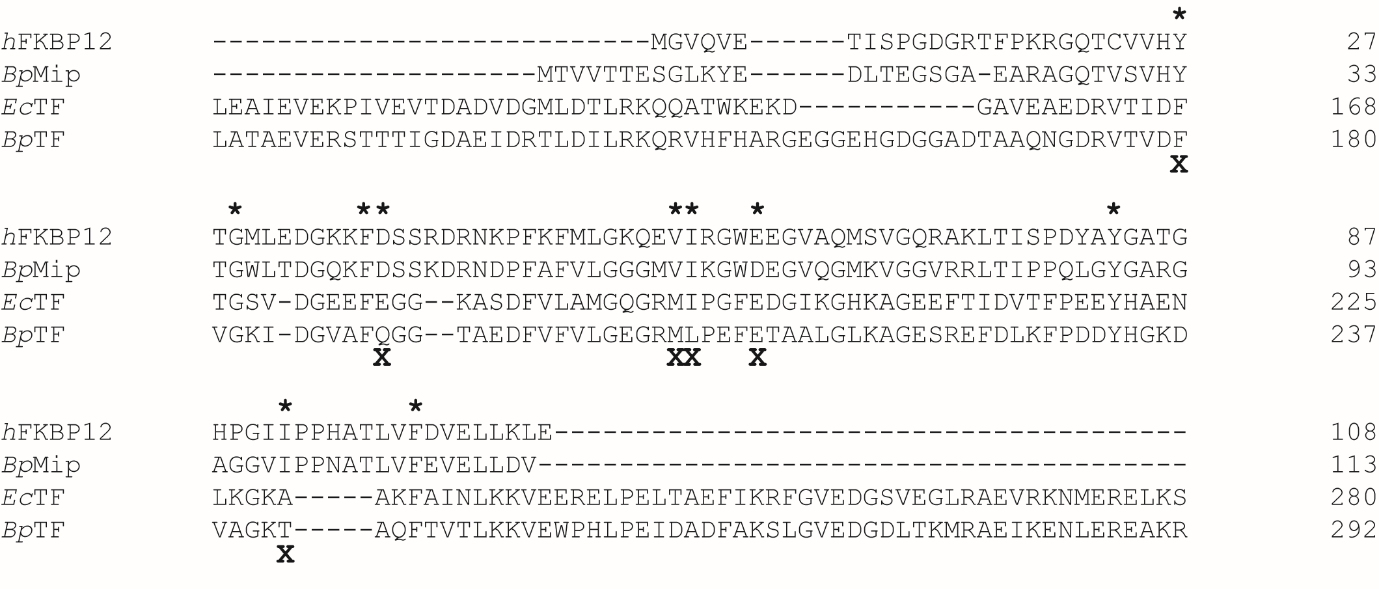
**FIG S4 Sequence analysis of *Bp*TF against *h*FKBP12 showed a lack of conservation of the amino acid residues important for macrolide binding.** The amino acid sequences of *h*FKBP12 (P62942/FKB1A_HUMAN), BPSS1823 (*Bp*Mip) (Q63J95_BURPS), *Ec*TF (P0A850/TIG_ECOLI) and BPSL1402 (*Bp*TF) (Q63V42/TIG_BURPS) were obtained from UniProt and aligned using Clustal Omega multiple sequence alignment tool. BPSS1823 (*Bp*Mip) and *Ec*TF were used as a control bacterial FKBPs that are inhibited and uninhibited by rapamycin, respectively. The amino acids residues that are important for macrolide binding (1) are denoted by asterisk with the ones denoted by the x symbol not conserved in *Bp*TF.


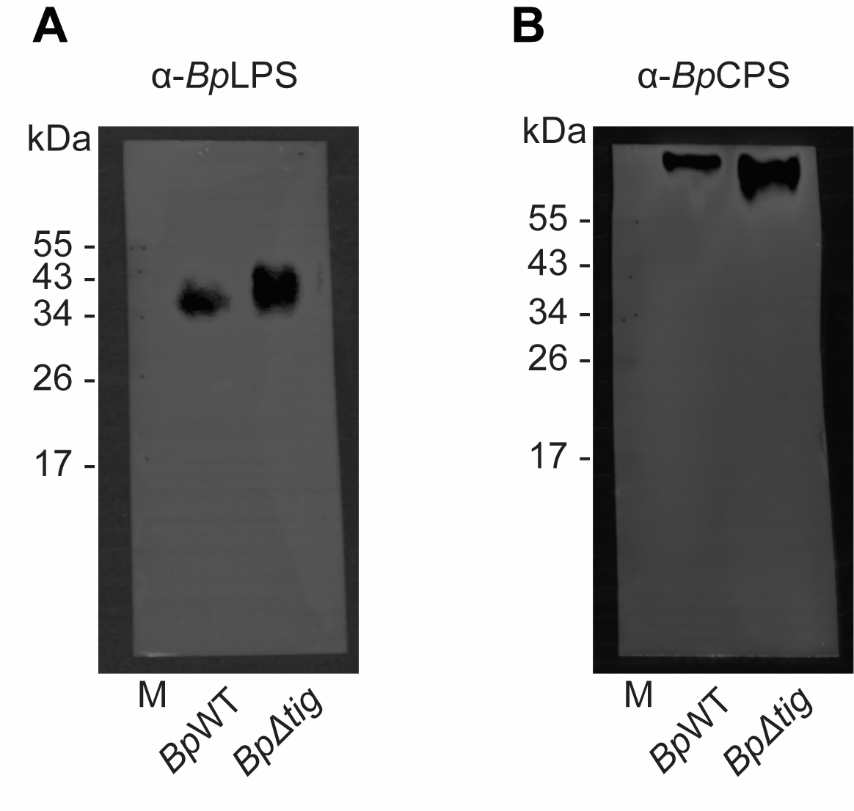


**FIG S5** Polysaccharide analysis of the *Bp∆tig* mutant. Overlay images captured by the UVITEC Alliance Q9 Advanced imager of immunoblots of proteinase K treated whole cell extracts of *Bp*WT and *Bp∆tig* strains developed using the (A) anti-*B. pseudomallei* LPS and (B) anti-*B. pseudomallei* CPS antibody. Lane M: Blue Prestained Protein Standard, Broad Range (11-250 kDa); Lane 2, *Bp*WT; Lane 3, *Bp∆tig*.

**References**

1. Stoller G, Tradler T, Rücknagel KP, Rahfeld JU, Fischer G. An 11.8 kDa proteolytic fragment of the E. coli trigger factor represents the domain carrying the peptidyl-prolyl cis/trans isomerase activity. FEBS Lett. 1996;384(2):117-22.
